# The Fragile First Year: GPS Tracking Identifies Post‐Release Survival Risks in Migratory Geese

**DOI:** 10.1002/ece3.71725

**Published:** 2025-07-04

**Authors:** Chao Zhang, Chaoyang Wang, Jiming Cheng, Yingqun Feng, Zhenyu Wang, Qiang Wang, Yankuo Li

**Affiliations:** ^1^ College of Life Sciences Jiangxi Normal University Nanchang Jiangxi China; ^2^ School of Life Sciences Central China Normal University Wuhan Hubei China; ^3^ Key Laboratory of Wetland Ecology and Environment, Northeast Institute of Geography and Agroecology Chinese Academy of Sciences Changchun Jilin China

**Keywords:** behavioral factors, environmental factors, GPS tracking, migratory geese, survival analysis

## Abstract

Migratory birds play a critical role as sentinels of ecosystem health, serving as key indicator species for monitoring biodiversity changes and environmental conditions. The survival of migratory birds has always been a focus of attention, and the first year following post‐release represents a critical period for establishing stable survival outcomes. However, it is difficult to track the life status of migratory birds, because some drivers affecting their survivorship may be hard to identify. Here, we investigated the survival and adaptation of Tundra Bean Geese (*Anser serrirostris serrirostris*) and Greater White‐fronted Geese ( *A. albifrons* ) by using GPS tracking data in order to systematically assess how study design parameters, key behavioral traits, and environmental conditions influence the first‐year survival rates of migratory geese from post‐release to spring migration onset. We found that the first‐year survival probability was significantly affected by factors such as average daily activity levels and weather conditions, particularly wind speed. Notably, tracking device type also significantly influenced survival outcomes, with neckband tag units demonstrating superior performance compared to backpack tags. Contrary to expectations, geographic clusters showed no consistent survival times across study cohorts. These findings highlight the influences of study design parameters (particularly device selection), individual behavior, and local weather conditions on waterfowl survival time. Our results provide operational guidelines for GPS tracking deployment in geese conservation and are crucial for developing effective conservation strategies and management measures.

## Graphical abstract

This study investigates how factors such as behavior, weather conditions, and other variables influence the first‐year survival of Bean Geese and Greater White‐fronted Geese after release. We found that the average daily activity level from release to migration onset and local weather conditions before migration, especially wind speed, significantly affected the first‐year survival rate. Our findings provide insights into the adaptive strategies of migratory birds and the impact of environmental factors on their survival.

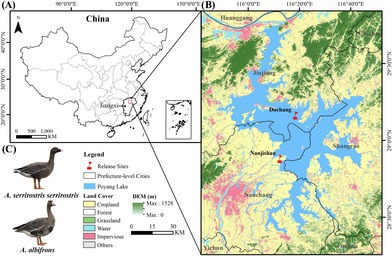

## 1 Introduction

Migratory birds are highly sensitive to environmental changes and have long served as important indicators for assessing ecosystem health (Van Doren et al. 2021; Zöckler 2005). Their complex life histories, which are characterized by long‐distance movements, habitat shifts, and physiological stress, make them model organisms for studying the ecological and behavioral adaptations necessary for survival (Viana et al. 2016; Wang et al. 2024; Zöckler 2005). Studies on migratory birds not only enhance our understanding of their adaptive strategies but also provide insights into the broader implications of environmental changes, including habitat loss, climate variability, and human disturbances (de Zwaan et al. 2022; Liang et al. 2021). Understanding the factors that influence their mortality, particularly during critical life stages, is essential for conservation planning. Yet, despite growing interest in avian survival, the interplay between individual condition, behavior, and local environmental conditions remains poorly understood, especially during the vulnerable period following release or marking.

The physiological condition, behavior, and environmental factors of birds before migration may influence their subsequent survival. Birds must replenish energy stores, regain normal activity patterns, and acclimate to the local environment, all while coping with potential stress from tagging and relocation (Bairlein and Simons 1995; Mandal et al. 2021; Tsvey 2023). Previous studies suggest that pre‐migratory conditions such as daily activity levels, weather, and habitat quality can influence the timing of departure, energy balance, and ultimately, survival outcomes (Paxton and Moore 2015; Rotics et al. 2021). For example, earlier migration is often associated with higher reproductive success and better survival prospects (Rotics et al. 2018). Conversely, failure to adequately prepare for migration may lead to premature mortality, especially in birds weakened by tagging or environmental stressors (Newton 2007). Despite this, the links between pre‐migratory traits and subsequent survival remain underexplored, particularly in wild populations tracked from the moment of release. Importantly, research design parameters such as species identity, release site characteristics, source population, and tracking device specifications can also shape survival outcomes and must be carefully considered in interpetation. Species‐specific traits, such as body size, migratory strategy, and ecological niche, determine both the suitability of tracking devices and the ecological relevance of release sites. For example, species with greater environmental adaptability may exhibit higher survival under post‐release stress (Dunbar 2020). Additionally, the type of tracking device used can influence behavior and survival: neckband tags and backpack harnesses have been shown to differentially impact flight dynamics and energy expenditure in large‐bodied waterbirds (Kölzsch, Neefjes, et al. 2016). These methodological choices are more than logistical concerns, as they directly influence animal performance and survival, particularly in the vulnerable window following tagging. Despite advancements in technology that have provided valuable data on bird migration, much research has focused primarily on migration stages that have already begun, overlooking the critical importance of the pre‐migration period, especially for birds tracked for the first time (Bonnet‐Lebrun et al. 2020; Yu 2023). This early phase, when individuals face novel environments and potential handling effects, remains under‐investigated and may hold key insights into survival dynamics.

Research on animal survival and its influencing factors has long been a key focus in conservation management. However, this field has historically been limited by technological constraints and the difficulty of acquiring data. The exact time of death is often unknown because unmarked individuals cannot be monitored closely over time. Recent advances in GPS tracking and survival analysis have revolutionized our ability to monitor individual birds with high temporal and spatial resolution (Cope et al. 2022; Hulthén et al. 2022; Gould et al. 2024; Lisovski et al. 2024; Sanz‐Aguilar et al. 2012; Scholer et al. 2020; Yanco et al. 2024). These tools have shed light on migratory strategies, habitat use, and mortality patterns across diverse avian taxa (Bussolini et al. 2024; Chan et al. 2024; Krenhardt et al. 2024; Schindler et al. 2024; Ugland et al. 2024). However, most studies have focused on survival during active migration or breeding, while overlooking the crucial pre‐migration stage, especially the period immediately following release or initial tagging. For birds fitted with tracking devices for the first time, this early period may be especially critical, as they must recover physiologically, adapt behaviorally, and respond to unfamiliar environmental conditions. To address this gap, the present study investigates how early post‐release behavior, habitat use, and local weather conditions affect first‐year survival in two migratory goose species. We also assess the influence of study design parameters such as species, release site, source population, and tracking device type, thereby integrating both biological and methodological considerations into our survival analysis framework.

Poyang Lake, located in Jiangxi Province, China, is the largest freshwater lake in the country and a globally important wetland. It is a critical wintering ground for hundreds of thousands of migratory waterbirds, including Siberian Crane (*Leucogeranus leucogeranus*), White‐naped Crane ( *Grus vipio* ), and Hooded Crane ( *G. monacha* ) (Zhang et al. 2022). The lake's unique hydrological regime, characterized by seasonal water level fluctuations, supports diverse habitats essential for the survival of waterbirds (Duan et al. 2023; Yang et al. 2020). As part of the East Asian–Australasian Flyway, Poyang Lake provides an ideal natural laboratory for studying the interaction between migratory birds and their habitats under different environmental conditions (Wang et al. 2019). Among the numerous species that winter in the lake, Tundra Bean Geese (*Anser serrirostris serrirostris*) and Greater White‐fronted Geese ( *A. albifrons* ) are particularly abundant and widely distributed across the Poyang Lake region, making them ideal species for studying migratory behavior and survival. These species exhibit diverse survival strategies, including variations in migratory timing, routes, and stopover site use, making them ideal candidates for studying inter‐individual differences in migration and survival (Lei et al. 2019; VonBank et al. 2024; Zhong et al. 2022). Tundra Bean Geese and Greater White‐fronted Geese also have a wide distribution across Eurasia; both species rely heavily on wetland ecosystems and are considered sensitive indicators of environmental change, including climate variability and habitat degradation (Amat and Green 2010; Musilová et al. 2018; Pavon‐Jordan et al. 2019, 2020; Wang et al. 2021).

In this study, we used high‐resolution GPS tracking data and survival analysis to examine the first‐year survival of Tundra Bean Geese and Greater White‐fronted Geese released at Poyang Lake. Our primary goal was to identify the key drivers of the post‐release to spring migration onset. We focused on factors such as habitat use (home range size), duration between release and migration initiation, weather conditions (e.g., wind speed and temperature), and daily activity patterns. We also evaluated how study design elements such as species identity, source population, release location, release year, and tracker type influenced survival outcomes. Our results provide new insights into how intrinsic traits and extrinsic environmental factors shape migratory bird survival. This work contributes to the refinement of GPS‐based monitoring protocols and offers practical recommendations for the conservation of wetland habitats and the management of migratory bird populations.

## 2 Materials and Methods

### 2.1 Study Area

**FIGURE 1:**
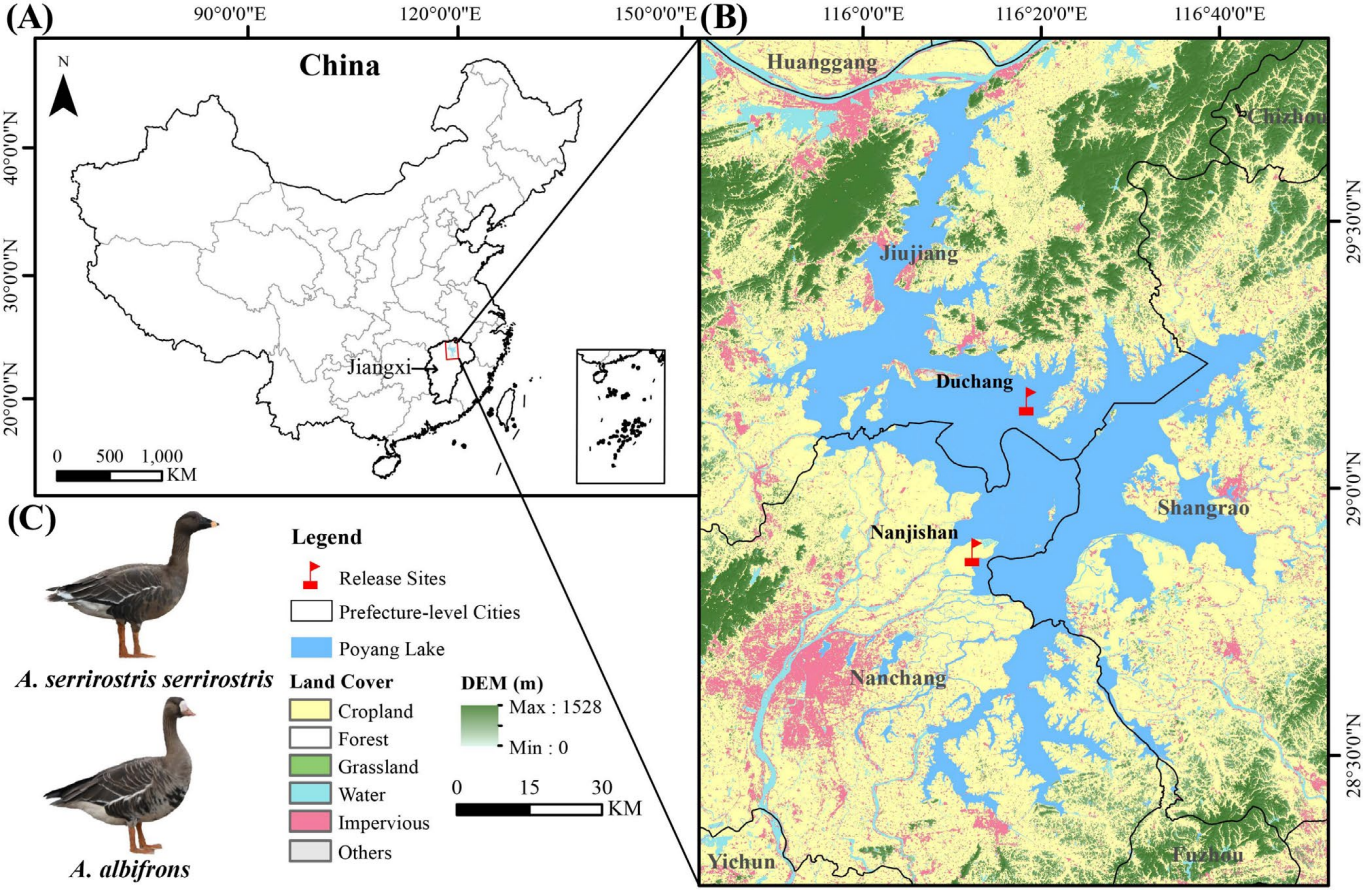
(A) The location of Poyang Lake in China. (B) The sampling and release sites for the two geese species (Duchang and Nanjishan), along with information on land use in and around Poyang Lake. (C) Photos of Tundra Bean Geese and Greater White‐fronted Geese are provided for reference.

This study was conducted in Poyang Lake (28°22′–29°45′ N, 115°47′–116°45′ E; Figure 1A,B), China's largest freshwater lake (Feng et al. 2015). Serving as a critical wetland nexus for both the East Asian‐Australasian Flyway (EAAF) and Central Asian Flyway (CAF) (Song et al. 2024; Sun et al. 2020), this ecosystem sustains over 400,000 migratory waterbirds annually. The wintering assemblage includes more than 300,000 Anatidae individuals, comprising key species such as Tundra Bean Geese, Greater White‐fronted Geese, Swan Geese ( *A. cygnoides* ), and Greylag Geese ( *A. anser* ) (Li et al. 2019). The region features a dynamic hydrological regime characterized by seasonal water level fluctuations, creating a mosaic of habitats such as shallow wetlands, mudflats, and grasslands (Hu et al. 2010; Yang et al. 2020). These habitats are essential for migratory birds, providing food resources and shelter during the winter months.

### 2.2 Source of Study Individuals

The study focused on two goose species, Tundra Bean Geese and Greater White‐fronted Geese (Figure 1C). Based on the annual waterbird survey data, it is conservatively estimated that during the wintering period, the populations of these two species account for more than 30% of the total waterbird numbers in Poyang Lake. The subjects come from two key areas: Duchang Migratory Bird Nature Reserve and Nanji Wetland National Nature Reserve of Poyang Lake, ensuring a representative sample that reflects the diversity in life histories and environmental exposures of these geese (Table 1). The study individuals originated from two sources: (1) wild geese captured during overwintering periods for waterbird epidemic surveillance, and (2) rehabilitated individuals released by the local wildlife management department after being rescued primarily due to injuries (e.g., collision trauma). All procedures were approved by the Ethics Committee of the College of Life Sciences, Jiangxi Normal University, and conducted under the supervision of staff from two protected areas where the captures occurred. Wild geese were captured at night using dim red lights and breathable cotton bags directly in their roosting habitats, following the methods described in Guidelines to the Use of Wild Birds in Research (Fair et al. 2010). All operations were performed by experienced reserve personnel to minimize handling stress and potential harm to the birds. A total of 37 individuals (26 *A. serrirostris serrirostris* and 11 *A. albifrons* ) were included in this study. All individuals were weighed. Sex and precise age determination proved challenging for non‐breeding individuals; morphological characteristics confirmed all as adult geese. Wild‐caught individuals underwent immediate health screening by reserve staff, while rehabilitated individuals were confirmed fully recovered prior to inclusion.

**TABLE 1:** Summary of study sample characteristics (total *N* = 37).

Characteristic	Category	*N* (%)
Release site	Duchang	20 (54.1)
	Nanjishan	17 (45.9)
Source type	Capture	30 (81.1)
	Rescue	7 (18.9)
Tracker type	Neck	17 (45.9)
	Backpack	20 (54.1)
Species	*A. fabalis*	25 (67.6)
	*A. albifrons*	12 (32.4)
Release year	2019	25 (67.6)
	2022	12 (32.4)

### 2.3 Deployment of Tracking Devices

All tested geese were fitted with either neckband or backpack GPS tracking devices, with the selection being random. Geese were acquired during two periods, in the spring of 2019 and 2022 (Table 1). The GPS tracking devices used in this study (Table S1) were manufactured by Hunan Global Messenger Technology Co. Ltd., including three models: HQNG4625S (neckband), HQBG3621S (backpack), and HQNG4625P (neckband). The backpack tags weighed 23 g (HQBG3621S), and the two neckband tags weighed 30 g (HQNG4625S) and 34 g (HQNG4625P); all of the devices were less than 3% of the average body mass of study geese (3.0 kg), minimizing potential impacts on natural behavior (Kenward 2000). Following device attachment, each individual underwent a brief observation period strictly limited to: (1) verifying proper transmitter function through signal confirmation, and (2) monitoring immediate post‐handling responses to ensure safe release capability. Animals displaying normal locomotor function and alert behavior (≤ 20 min observation) were prioritized for immediate release at designated Poyang Lake sites. This protocol followed ARRIVE guidelines for wildlife telemetry studies, balancing data validity with stress minimization through standardized quick‐release criteria. All individuals were remotely monitored via satellite tracking to identify any signs of abnormal movement patterns or potential device‐related injuries.

### 2.4 Tracking Data Collection

The GPS tracking data were extracted from the Global Messenger Satellite Tracking Data Service Platform. The GPS trackers employed GSM‐based communication systems for real‐time data transmission and were equipped with solar panels to ensure continuous power supply, enabling hourly location fixes and synchronized data retrieval. The GPS positioning accuracy of the satellite trackers is classified into six levels: A (±5 m), B (±10 m), C (±20 m), D (±100 m), E (±2000 m), and invalid (unable to locate). For data analysis, only data with accuracy levels A–C were considered. The dataset includes 10 variables: longitude, latitude, speed, heading, altitude, exercise volume, temperature, time, voltage, and positioning accuracy. Exercise volume is defined as the number of times the device moves within a collection cycle (1 h). The movement quantification is based on overall dynamic body acceleration (ODBA), which integrates dynamic acceleration components from all three axes (*X*, *Y*, and *Z*) while excluding static gravitational forces. The accelerometer continuously monitors triaxial acceleration data, calculating ODBA as the sum of absolute dynamic acceleration values across all axes. When the computed ODBA value exceeds the predetermined threshold of 0.15 G (where 1 G = 9.8 m/s^2^), indicative of substantial body movement, the movement counter increments by one count. The ODBA threshold was determined based on calibration protocols provided by the device manufacturer (Hunan Global Messenger Technology Co. Ltd.), effectively distinguishing baseline activity from significant motion events.

### 2.5 Environmental and Weather Data

Environmental and weather data were collected to evaluate their influence on survival. Weather variables include daily temperature (TEMP), dew point (DEWP), sea‐level pressure (SLP), station pressure (STP), visibility (VISIB), wind speed (WDSP), maximum wind speed (MXSPD), maximum temperature (MAX), minimum temperature (MIN), and precipitation (PRCP), which were sourced from the National Centers for Environmental Information (NCEI) database (https://www.ncei.noaa.gov/). Meteorological data from Nanchang Changbei International Airport station (28°51′ N, 115°54′ E) were selected following proximity analysis to our study area. Comparison with candidate stations (Lushan, Jingdezhen) using Euclidean distance calculations in ArcMap 10.6 identified Nanchang as both the closest station to the Poyang Lake shoreline (≈14.3 km NW) and the nearest to all recorded bird activity sites. We calculated daily averages of environmental variables during the 14‐day period preceding each individual's first northward migration departure from Poyang Lake. These temporal averages characterized pre‐migration exposure profiles, serving as predictive covariates in survival analyses. Nanchang's data remain spatially relevant given our focus on local conditions immediately preceding migration initiation from this region.

### 2.6 Statistical Analysis

The timing of the first northward migration and the duration between release and migration initiation (StayDur) were determined for each individual using data transmitted from GPS tracking devices. Additionally, the average daily activity levels (ActAvg) from post‐release to spring migration onset were calculated based on the accelerometer data provided by the tracking devices. The sum of the hourly activity values over a 24‐h period equals the average daily activity levels.

Home range and core area sizes during the period from tagging to the onset of the first migration were estimated using Kernel Density Estimation (KDE) in ArcMap 10.6, with 95% KDE representing home range and 50% KDE denoting core areas. These metrics were derived to assess spatial use patterns. In survival analyses, the 95% kernel density isopleth (KDE) was employed to assess environmental exposure covariates across the full utilization distribution, whereas the 50% KDE core areas were specifically analyzed through Moran's *I* spatial autocorrelation to capture intensive space use patterns.

Hierarchical clustering based on goose locations was conducted using the *stats* (R Core Team 2024), *dendextend* (Galili 2015), and *circlize* (Gu 2014) packages for visualizing and customizing dendrograms. This aimed to test the hypothesis that individuals sharing similar spatial domains would exhibit comparable survival times. To explore spatial clustering of survival data, Global Moran's *I* analysis was performed in ArcMap 10.6 using home range data derived from 50% KDE values. It was employed to detect potential spatial autocorrelation in survival time data, which could indicate unmeasured environmental covariates (e.g., hydrological conditions or predator density) influencing mortality risks across the landscape.

We first excluded hunting as the cause of mortality by identifying characteristic patterns in movement data. Features associated with hunting were defined as sudden signal loss or abrupt cessation of movement. Mortality events were classified based on gradual declines in acceleration (ODBA), altitude, and movement velocity during the days preceding death, indicating physiological decline rather than sudden external intervention (i.e., hunting). Survival time was defined as the time interval (days) from initial tagging to biologically confirmed mortality, determined by simultaneous fulfillment of three criteria: (1) continuous 72‐h thermal equilibration (±2°C difference between body temperature sensor and ambient environment), (2) sustained behavioral quiescence (< 10 accelerometer counts per hour maintained for ≥ 24 consecutive hours), and (3) persistent spatial confinement within a 200 m radius for ≥ 72 h preceding transmitter signal termination. This method was established based on our previous research experience and by referencing relevant literature (Sergio et al. 2019). Individuals surviving beyond 365 days were right‐censored with survival time truncated at 365 days. To analyze survival, we primarily employed Kaplan–Meier (KM) survival analysis and multivariate Cox proportional hazards analysis.

As a statistical method, survival analysis has been widely used in medicine, epidemiology, and ecology, as well as assessing factors influencing the time until the occurrence of an event such as death or failure (D'Arrigo et al. 2021; Grosbois et al. 2008). This method has been proven to be essential for understanding mortality and survival dynamics in both human and animal populations, providing insights into the causes of death and identifying key risk factors (Bustamante‐Teixeira and Faerstein 2002; D'Arrigo et al. 2021). Common survival analysis models include parametric (e.g., exponential, Weibull), non‐parametric (Kaplan–Meier), and semi‐parametric approaches (Cox regression). We employed Kaplan–Meier survival analysis and Cox proportional hazards models. Kaplan–Meier survival analysis is a non‐parametric method used to estimate survival probabilities over time. It provides a stepwise survival curve, where each step corresponds to an event (e.g., mortality or signal loss). The survival probability at any time 𝑡 is calculated as:

(1) $$s\left(t\right)=-\prod_{1:t_{i}\leq t}\left(1-{\mathrm{34𝑑}}_{i}/n_{i}\right)$$

where 𝑡_𝑖_ represents the time of the 𝑖th event, 𝑑_𝑖_ is the number of events occurring at 𝑡_𝑖_, and 𝑛_𝑖_ is the number of individuals at risk just before 𝑡_𝑖_. Kaplan–Meier analysis is particularly useful for comparing survival curves between groups, and differences can be statistically tested using the log‐rank test (Kaplan and Meier 1958).

The Cox proportional hazards model is a semi‐parametric regression method that assesses the relationship between covariates and survival time. It assumes that the hazard ratio (HR) between groups remains constant over time (proportional hazards assumption), where HR > 1 indicates higher risk in the exposure group compared to the reference group, HR < 1 suggests lower risk, and HR = 1 implies no difference in risk. The hazard function is expressed as:

(2) $$h\left(t|X\right)=h_{0}\left(t\right)\exp\left(\beta_{1}X_{1}+\beta_{2}X_{2}+\cdots +\beta_{p}X_{p}\right)$$

where ℎ(𝑡∣𝑋) is the hazard at time 𝑡 given covariates 𝑋 = (𝑋_1_, 𝑋_2_, …, 𝑋_𝑝_), ℎ_0_(𝑡) is the baseline hazard function, and 𝛽_1_, 𝛽_2_, …, 𝛽_𝑝_ are coefficients estimating the effect of covariates on the hazard. This model identifies factors that significantly influence survival time, allowing for simultaneous adjustment of multiple variables (Cox 1972).

Kaplan–Meier analysis was used to exclude the influence of experimental factors (including species, release sites, sources, tracking device types and release year) on the survival of the study subjects. Cox proportional hazards regression was applied to assess the effects of study design, physiological, behavioral, and weather‐related factors on survival time, with Schoenfeld residuals tested using the *survival* and *survminer* (Kassambara et al. 2021) packages to confirm the proportional hazards assumption. We performed a forward stepwise Cox regression analysis to identify significant factors influencing survival time. Prior to the regression analysis, multicollinearity among variables was assessed, and variables with a variance inflation factor (VIF) greater than 5 were excluded (Johnston 1988). To identify the most significant factors influencing survival time, we performed a comprehensive all‐subsets regression analysis to evaluate all possible combinations of covariates, excluding study design variables. Model selection was guided by the Akaike information criterion (AIC), where all competing models with ΔAIC < 2 were retained for multimodel inference. The final model averaging procedure prioritized the most parsimonious configuration among equally plausible candidates.

All statistical analyses were performed in R (version 4.4.0) and ArcMap 10.6. Kaplan–Meier survival analysis was conducted using the *survival* package (Therneau 2024), and survival curves were visualized with the *ggplot2* package (Wickham 2016). Data preprocessing and summary statistics were completed using the *dplyr* (Wickham et al. 2023) and *tidyr* (Wickham et al. 2024) packages, ensuring a streamlined workflow. All data visualization steps emphasized clarity and reproducibility through *ggplot2* and *ggtree* (Xu et al. 2022) packages.

## 3 Results

### 3.1 Kaplan–Meier Analysis of Study Design Factors

**FIGURE 2:**
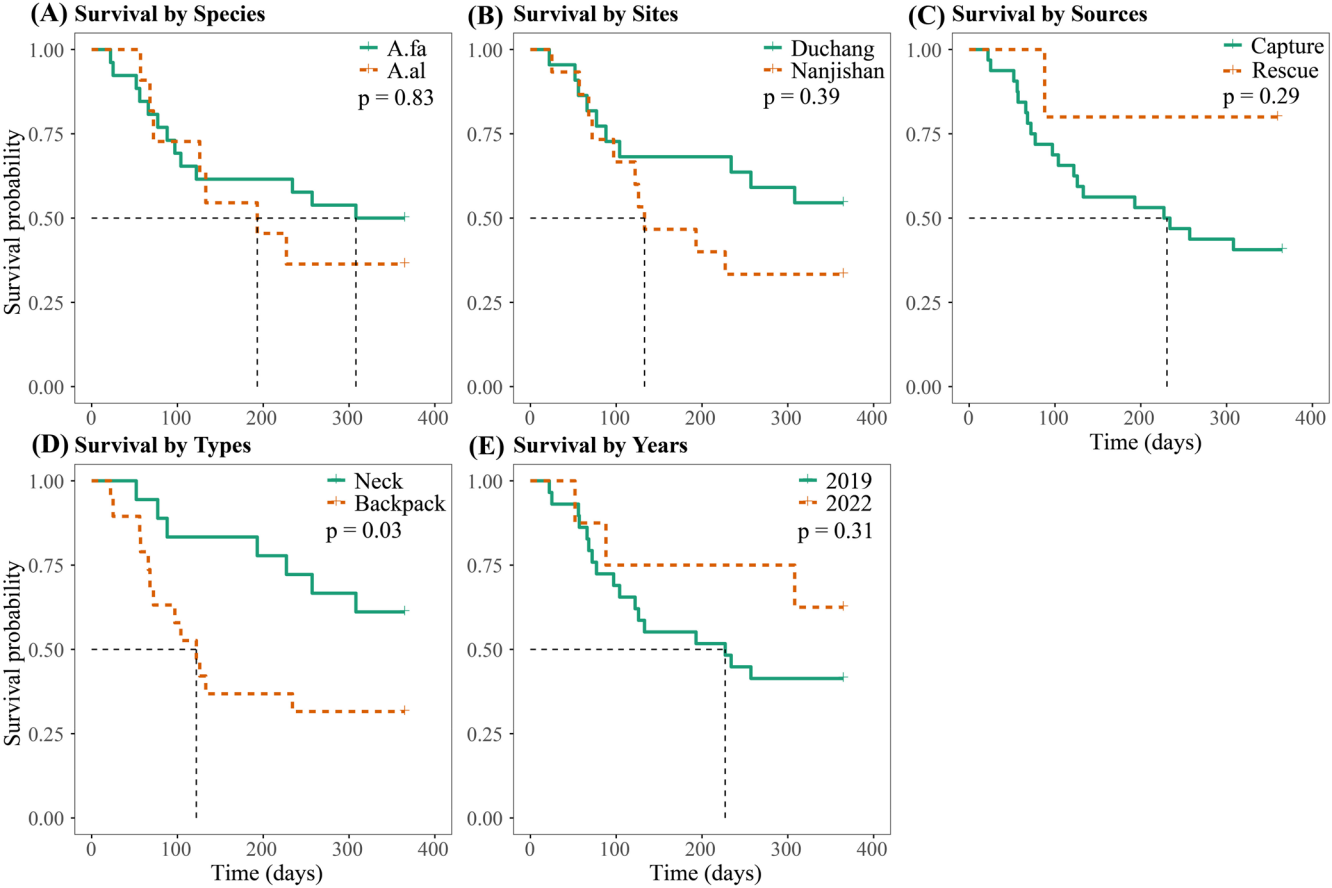
Kaplan–Meier survival curves for different experimental factors, including species (A), release sites (B), sources (C), tracker types (D), and release years (E). Solid green and dashed orange lines represent the estimated survival probabilities for each group based on the Kaplan–Meier survival curves. Dashed vertical lines indicate median survival time (time at which 50% survival probability is reached).

Kaplan–Meier survival curves were generated to evaluate survival probabilities based on species, release sites, tracking device types, sources of geese, and release years. The survival rate of geese decreased rapidly in the first year after the post‐release. The results indicated no statistically significant differences in survival probabilities across species (log‐rank test: *χ* ^2^ = 0.05, df = 1, *p* = 0.83), release sites (log‐rank test: *χ* ^2^ = 0.73, df = 1, *p* = 0.39), or sources (log‐rank test: *χ* ^2^ = 1.13, df = 1, *p* = 0.29). However, a significant difference was observed between tracking device types (log‐rank test: *χ* ^2^ = 4.58, df = 1, *p* = 0.03), with neckband tags showing higher survival probabilities compared to backpack tags. Similarly, no significant differences were observed between the years of release (log‐rank test: *χ* ^2^ = 1.04, df = 1, *p* = 0.31) (Figure 2A–E).

### 3.2 Spatial Survival Correlations

**FIGURE 3:**
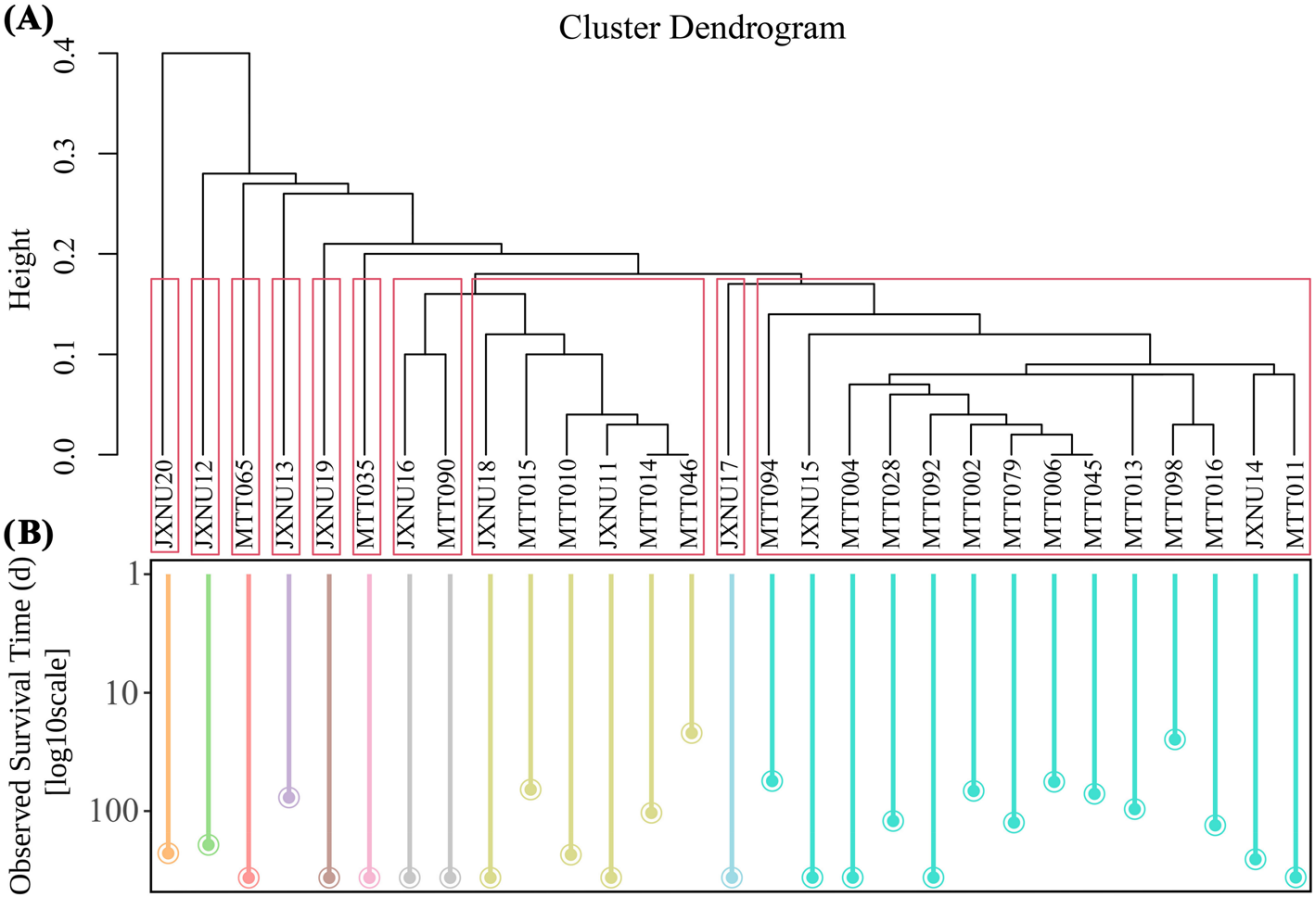
(A) The dendrogram of distance‐based clustering for individual geese based on their distribution sites before the first migration. (B) The survival time (log_10_‐transformed) for each individual as of the analysis date, with distinct clusters differentiated by color coding in the graphical representation.

Based on the clustering analysis of tracking locations for individual geese, the hierarchical dendrogram showed groups of individuals with similar spatial patterns of site use. However, individuals within the same cluster group did not demonstrate consistent survival times, indicating that survival time was not associated with clustering patterns (Figure 3A,B). Furthermore, Moran's *I* spatial autocorrelation analysis, conducted using the 50% kernel density estimates (50% KDE) of individual home ranges, revealed no significant spatial autocorrelation (Moran's *I* = −0.046, *p* = 0.869).

### 3.3 Survival Determinants From Cox Regression

**FIGURE 4:**
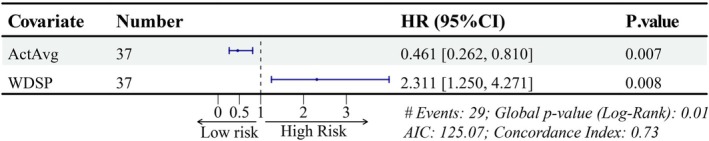
Results of multivariate Cox regression analysis. ActAvg, average daily activity levels before the first northward migration; WDSP, wind speed.

After removing multicollinearity, the variables Releasetime, StayDur, ActAvg, KDE, Weight, VISIB, WDSP, TEMP, and PRCP were ultimately included in the initial model. The final model retained two key predictors: ActAvg and WDSP (Table S2). The results demonstrate that ActAvg had a significant positive effect on survival (HR = 0.461, 95% CI: 0.262–0.810, *p* = 0.007), indicating that geese with higher pre‐migration activity (primarily foraging behavior) had lower survival risks. On the other hand, WDSP showed a significant negative effect on survival (HR = 2.311, 95% CI: 1.250–4.271, *p* = 0.008), suggesting that higher wind speeds were linked to increased survival risks. Additionally, the overall model demonstrated a good fit (global *p*‐value [log‐rank] = 0.005776; AIC = 163.15; concordance index = 0.72), suggesting the model effectively captured survival‐related factors (Figure 4).

## 4 Discussion

This study provides valuable insights into the survival dynamics of migratory geese following release with GPS tracking devices. Through survival analysis, cluster analysis, and Cox regression modeling, we evaluated the influence of experimental, physiological, behavioral, and environmental factors on post‐release survival. The results highlight both key predictors of survival and the importance of study design in reintroduction outcomes.

### 4.1 Effects of Study Design Factors on Geese Survival

Among the experimental design factors, most showed no significant effect on post‐release survival. The lack of significant interannual variation in survival aligns with expectations for K‐selected species like geese, where adult survival rates are typically buffered against environmental fluctuations due to their long lifespans and delayed reproduction strategies (Forrester et al. 2024). This is further supported by environmental monitoring data from Poyang Lake National Nature Reserve annual reports, which documented no extreme climatic events (e.g., severe droughts or floods) during the wintering periods preceding our study years (Liu et al. 2020; Xu et al. 2023). Similarly, we found no significant differences in survival between species, release sites, or sources. The species‐level similarity may be attributed to the close taxonomic relationship between the two goose species, which share similar morphological and ecological adaptations (Ottenburghs et al. 2016). The absence of site‐specific effects contrasts with studies on other waterfowl where release site characteristics strongly influenced post‐release survival (Lloyd et al. 2019; Ruzicka et al. 2024), potentially reflecting the homogeneous habitat quality across our study sites or rapid environmental acclimatization by geese.

However, tracking device type had a significant effect on survival: geese fitted with neckband tags had higher survival rates than those with backpack tags (log‐rank test: *χ* ^2^ = 4.58, df = 1, *p* = 0.03), with neckband tags showing higher survival probabilities compared to backpack tags. This finding resonates with recent biomechanical studies demonstrating that dorsal‐mounted backpack devices substantially alter avian aerodynamics by shifting the center of mass and increasing drag coefficients, thereby elevating flight energy expenditure (Brlík et al. 2020; Katzner and Young 2024; Pennycuick et al. 2012; Thaxter et al. 2016). A study by Mizrahy‐Rewald et al. (2023) on Northern Bald Ibises demonstrated that back‐mounted trackers (secured via wing loops above the back) significantly reduced their migratory flight distance. The neckband design used in our study offers several biomechanical advantages over backpack‐style tags. Unlike backpacks, neckbands avoid contact with wings and major muscle groups, thereby reducing aerodynamic drag and minimizing interference with flight mechanics. This design also prevents constriction of the thoracic cavity, which may be critical during periods of intense metabolic activity, such as pre‐migration staging and sustained flight. While initial behavioral impacts of neckband tags (e.g., increased preening) typically subside within 6 days post‐deployment (Clausen et al. 2020), backpack devices may impose chronic physiological costs through altered gait mechanics and feather abrasion during prolonged use (Lamb et al. 2020). It is theoretically plausible that backpack‐style harnesses may restrict thoracic expansion during hyperphagic periods prior to migration, potentially limiting fat accumulation and energy reserves. Although our study did not directly assess this hypothesis, future research could explore this mechanism through comparative studies of pre‐migration body condition in tagged versus untagged birds. This could help clarify the physiological costs associated with different tagging methods during critical staging periods. Although our study provides evidence that neckband tags are associated with higher post‐release survival compared to backpack tags, it is important to note that this conclusion is based on a relatively modest sample size (17 neckband, 20 backpack). While consistent with prior biomechanical research, these findings should warrant replication in future studies with larger sample sizes and additional species to assess potential interspecific variation in response to device type.

Research on Pink‐footed Geese has demonstrated temporal sensitivity in tracker effects (Schreven et al. 2024). Device positioning during critical life history stages may disproportionately impact fitness outcomes. While our neckband tags design (< 3% body mass) adheres to recommended mass thresholds, this threshold may overlook the critical role of tag shape and placement (Katzner and Young 2024). The observed device effects underscore the imperative for longitudinal monitoring frameworks in avian telemetry research. Implanted transmitters in sea ducks were shown to disrupt movement patterns for ≥ 5 days post‐deployment and suppress breeding site fidelity across entire reproductive seasons (Lamb et al. 2020). While our 1‐year study captured acute survival impacts, it likely missed delayed reproductive trade‐offs. To mitigate adverse effects, future telemetry protocols should: (1) prioritize neckband tags deployment for Anseriformes GPS tracking, and (2) implement systematic monitoring of post‐marking reproductive performance throughout subsequent breeding cycles.

### 4.2 Factors Influencing Survival: Activity, Weather Conditions

Our Cox regression analysis revealed that behavioral and environmental variables played important roles in shaping survival. Notably, higher average daily activity levels from release until the first northward migration were positively associated with survival. This finding is consistent with other studies on migratory birds, where increased pre‐migration activity has been linked to higher survival probabilities (Rotics et al. 2021). This suggests that higher pre‐migration activity, possibly reflecting stronger foraging behaviors, may improve the birds' physical condition and thus increase survival chances. Wind speed was another significant factor, with higher wind speeds associated with an increased hazard ratio, indicating that stronger winds are detrimental to bird survival, which is in line with previous research (Huang et al. 2017; Loonstra et al. 2019). Severe weather, particularly high winds, increases the difficulty of migration or post‐release survival and also may exacerbate the risks faced by migratory birds during critical phases of their journeys. These results underscore the dual importance of intrinsic behavioral traits and extrinsic environmental pressures in determining post‐release outcomes.

### 4.3 Non‐Significant Factors and the Potential Role of Unmeasured Environmental Variation

The results of spatial clustering and Moran's I autocorrelation did not show any relationship between the survival of the studied individuals and their spatial locations. This suggests that the survival patterns of the studied individuals were not spatially structured, and factors beyond spatial proximity might play a more critical role in determining survival outcomes. Interestingly, no significant effects were found for StayDur, KDE, Weight, VISIB, TEMP, or PRCP on survival. This suggests that other factors, such as increasing foraging activity, may have played a more pivotal role in survival. Lopez‐Hervas et al. (2024) experimentally demonstrated that life‐history and personality traits adapt at different rates to environmental changes, further supporting this viewpoint. Additionally, the lack of significant effects for weather variables such as temperature and precipitation could reflect the birds' ability to adapt to varying environmental conditions, the limited variability in these factors during the study period, or the inherent lack of regulatory influence of these conditions on survival. For instance, it is possible that the temperature and precipitation during the study were not extreme enough to pose a significant threat to survival. This was also confirmed by the monitoring of Poyang Lake during the 2‐year experiment (Liu et al. 2020; Xu et al. 2023). Alternatively, birds may have developed coping mechanisms to handle these environmental factors. The absence of these factors as significant predictors could also point to the possibility that survival is more strongly influenced by dynamic behavioral and physiological responses, rather than static environmental variables. Additionally, although we observed no significant differences in survival across their spatial locations, this may not definitively exclude geographic influences. The environmental variables assessed may not fully capture finer‐scale heterogeneity in habitat structure, predation risk, or human disturbance. Moreover, we were unable to include individual sex and age as covariates, as these traits could not be reliably determined during the non‐breeding season. Future studies should consider molecular or long‐term tracking approaches to evaluate potential demographic effects on survival.

### 4.4 Implications and Future Directions

Unlike previous studies that primarily focused on long‐term migratory survival, our study emphasizes the critical post‐release period, highlighting how early behavioral responses can predict subsequent survival outcomes. We argue that although data covering the full life cycle of the geese are not used, physiological, behavioral, and environmental data from post‐release to spring migration onset still provide essential insights into the factors influencing geese's first‐year survival. These findings highlight that the early survival pressures primarily stem from insufficient food reserves and exposure to strong wind events. We identified three priority areas for improving reintroduction outcomes. First, neckband tags proved superior to backpack tags in post‐release monitoring. Second, sustained high activity levels through vigorous foraging emerged as the strongest predictor of survival, necessitating release sites with immediately available and replenishable food resources to meet acute energy demands. Third, exposure to strong winds increased mortality risk, highlighting the need for weather‐contingent release planning. We recommend the following strategies to enhance conservation outcomes: (1) use neckband tags as standard for geese telemetry; (2) verify food availability pre‐release and allow for emergency provisioning; and (3) avoid releasing birds during periods of predicted high wind speeds.

The negative impact of wind underscores the need to incorporate real‐time meteorological data in release planning. Previous research also suggests seasonality in wind effects, with autumn migration being especially vulnerable (Kölzsch, Müskens, et al. 2016). Geese may alter migration timing or routes in response to changing conditions (Clausen et al. 2018), and survival likely reflects a dynamic interplay between individual flexibility and external constraints. Further research should explore how behavioral plasticity mediates survival under fluctuating environmental conditions.

Our findings also offer practical implications for conservation programs operating across multiple regions. Given the geographic scope of the East Asian–Australasian Flyway and the increasing emphasis on coordinated species recovery efforts, our results may inform transboundary reintroduction strategies. For instance, aligning release timing with favorable weather windows and adopting standardized, minimally invasive tagging protocols may enhance survival outcomes at scale. These strategies could support the objectives of flyway‐level partnerships by improving consistency and efficacy in post‐release management. By integrating behavioral indicators such as pre‐migratory activity levels with environmental forecasting, practitioners can make more informed decisions to improve reintroduction success. Moreover, combining individual‐based tracking with regional habitat monitoring can facilitate adaptive conservation planning tailored to local and migratory contexts.

## 5 Conclusions

This study provides valuable insights into the survival dynamics of migratory geese after release with tracking devices. Our analysis demonstrates that post‐release survival is influenced by study design, behavioral traits, and environmental factors. Specifically, the selection of tracking devices is critical, with neckbands (e.g., GPS collars) being more suitable for geese. Additionally, higher pre‐migration activity levels were found to significantly improve survival rates, whereas adverse weather conditions, particularly strong winds, reduced survival probability.

The findings indicate that management efforts for birds undergoing initial tagging and release should prioritize monitoring behavioral patterns and environmental exposures during the critical window from post‐release to their first migration. Ensuring adequate resource availability (e.g., foraging habitats) and minimizing exposure to extreme weather events may enhance post‐release survival rates. This study underscores the multifaceted nature of survival dynamics in migratory geese, necessitating integrated monitoring frameworks that account for individual‐level behaviors and landscape‐scale environmental variables. Future research should investigate temporal and spatial interactions between meteorological conditions and survival outcomes to refine adaptive management protocols for migratory bird populations.

## Author Contributions

**Chao Zhang:** conceptualization (equal), data curation (equal), formal analysis (equal), methodology (equal), software (equal), visualization (equal), writing – original draft (equal). **Chaoyang Wang:** data curation (equal), investigation (equal). **Jiming Cheng:** writing – original draft (equal), writing – review and editing (equal). **Yingqun Feng:** methodology (equal), software (equal). **Zhenyu Wang:** investigation (equal), project administration (equal), resources (equal), supervision (equal), validation (equal). **Qiang Wang:** data curation (equal), funding acquisition (equal), project administration (equal). **Yankuo Li:** conceptualization (equal), data curation (equal), funding acquisition (equal), investigation (equal), project administration (equal), resources (equal), supervision (equal), validation (equal), writing – review and editing (equal).

## Ethics Statement

This work was carried out under the ethics permit Animal Ethics and Welfare Committee of the College of Life Sciences, Jiangxi Normal University, Ethical Review Acceptance Number: 20250115‐001.

## Conflicts of Interest

The authors declare no conflicts of interest.

## Supporting information


Table S1.
**Table S2**.

## Data Availability

The data that supports the findings of this study are available in the Supporting Information of this article.
