# Supplementary material for: The Fragile First Year: GPS Tracking Identifies Post‐Release Survival Risks in Migratory Geese
Source: Ecol Evol. 2025 Jul 4;15(7):e71725. doi: 10.1002/ece3.71725 (PMC12231202; doi:10.1002/ece3.71725)
Supplement: Supplementary file 1 — Table S1. Table S2. [file ECE3-15-e71725-s001.doc]

**SUPPORTING INFORMATION**

**Table S1.** Details of individual goose, including ID, release date, first migration date, body weight, release site, source, type of tracking device used, and species information.

| ID | Release date | First migration date | Weight (kg) | Release site | Source | Tracker type (model) | Species |
| --- | --- | --- | --- | --- | --- | --- | --- |
| JXNU11 | 2019/2/26 | 2019/3/20 | 3.1 | Duchang | Capture | Neck (HQNG4625S) | *A. fabalis* |
| JXNU13 | 2019/2/26 | 2019/3/26 | 2.1 | Duchang | Capture | Neck (HQNG4625S) | *A. fabalis* |
| JXNU14 | 2019/2/26 | 2019/4/6 | 3.6 | Duchang | Capture | Neck (HQNG4625S) | *A. fabalis* |
| JXNU15 | 2019/2/26 | 2019/3/12 | 3.1 | Duchang | Capture | Neck (HQNG4625S) | *A. fabalis* |
| JXNU16 | 2019/3/12 | 2019/4/24 | 3.2 | Duchang | Rescue | Neck (HQNG4625S) | *A. fabalis* |
| JXNU17 | 2019/2/26 | 2019/4/1 | 2.6 | Duchang | Capture | Neck (HQNG4625S) | *A. fabalis* |
| JXNU18 | 2019/2/26 | 2019/3/19 | 2.9 | Duchang | Capture | Neck (HQNG4625S) | *A. fabalis* |
| MTT010 | 2019/2/26 | 2019/3/14 | 3 | Duchang | Capture | Backpack (HQBG3621S) | *A. fabalis* |
| MTT011 | 2019/2/26 | 2019/3/31 | 2.1 | Duchang | Capture | Backpack (HQBG3621S) | *A. fabalis* |
| MTT013 | 2019/2/26 | 2019/4/6 | 3.2 | Nanjishan | Capture | Backpack (HQBG3621S) | *A. fabalis* |
| MTT014 | 2019/2/26 | 2019/3/15 | 3.2 | Duchang | Capture | Backpack (HQBG3621S) | *A. fabalis* |
| MTT015 | 2019/2/26 | 2019/3/15 | 3.2 | Duchang | Capture | Backpack (HQBG3621S) | *A. fabalis* |
| MTT028 | 2019/3/5 | 2019/3/17 | 2.8 | Nanjishan | Capture | Backpack (HQBG3621S) | *A. fabalis* |
| MTT035 | 2019/3/12 | 2019/4/5 | 3.1 | Duchang | Rescue | Backpack (HQBG3621S) | *A. fabalis* |
| MTT046 | 2019/2/26 | 2019/3/11 | 3.4 | Duchang | Capture | Backpack (HQBG3621S) | *A. fabalis* |
| MTT090 | 2019/3/12 | 2019/4/24 | 2.9 | Duchang | Rescue | Backpack (HQBG3621S) | *A. fabalis* |
| MTT094 | 2019/2/26 | 2019/3/10 | 3.5 | Duchang | Capture | Backpack (HQBG3621S) | *A. fabalis* |
| MTT098 | 2019/2/26 | 2019/3/11 | 3 | Nanjishan | Capture | Backpack (HQBG3621S) | *A. fabalis* |
| HQP5119 | 2022/3/1 | 2022/3/8 | 2.5 | Nanjishan | Capture | Neck (HQNG4625P) | *A. fabalis* |
| HQP5110 | 2022/3/9 | 2022/3/29 | 2.9 | Duchang | Capture | Neck (HQNG4625P) | *A. fabalis* |
| HQP5114 | 2022/3/9 | 2022/3/24 | 2.3 | Duchang | Capture | Neck (HQNG4625P) | *A. fabalis* |
| HQP5116 | 2022/3/9 | 2022/3/24 | 3.1 | Duchang | Capture | Neck (HQNG4625P) | *A. fabalis* |
| HQP5117 | 2022/3/9 | 2022/3/28 | 2.5 | Duchang | Capture | Neck (HQNG4625P) | *A. fabalis* |
| HQP5118 | 2022/3/9 | 2022/3/28 | 3 | Duchang | Capture | Neck (HQNG4625P) | *A. fabalis* |
| HQP5113 | 2022/3/15 | 2022/4/11 | 2.5 | Duchang | Rescue | Neck (HQNG4625P) | *A. fabalis* |
| HQP5115 | 2022/3/15 | 2022/5/5 | 2.5 | Duchang | Rescue | Neck (HQNG4625P) | *A. fabalis* |
| JXNU12 | 2019/2/26 | 2019/3/20 | 2.4 | Nanjishan | Capture | Neck (HQNG4625S) | *A. albifrons* |
| JXNU19 | 2019/2/26 | 2019/4/17 | 2 | Nanjishan | Capture | Neck (HQNG4625S) | *A. albifrons* |
| JXNU20 | 2019/2/26 | 2019/3/19 | 2.7 | Nanjishan | Capture | Neck (HQNG4625S) | *A. albifrons* |
| MTT002 | 2019/3/5 | 2019/3/19 | 2.3 | Nanjishan | Capture | Backpack (HQBG3621S) | *A. albifrons* |
| MTT004 | 2019/3/5 | 2019/4/1 | 2.1 | Nanjishan | Capture | Backpack (HQBG3621S) | *A. albifrons* |
| MTT006 | 2019/2/26 | 2019/3/19 | 2.2 | Nanjishan | Capture | Backpack (HQBG3621S) | *A. albifrons* |
| MTT016 | 2019/2/26 | 2019/5/2 | 2.7 | Nanjishan | Capture | Backpack (HQBG3621S) | *A. albifrons* |
| MTT045 | 2019/3/5 | 2019/3/26 | 2.3 | Nanjishan | Capture | Backpack (HQBG3621S) | *A. albifrons* |
| MTT065 | 2019/3/5 | 2019/4/5 | 2.3 | Nanjishan | Capture | Backpack (HQBG3621S) | *A. albifrons* |
| MTT079 | 2019/2/26 | 2019/4/1 | 2.4 | Nanjishan | Capture | Backpack (HQBG3621S) | *A. albifrons* |
| MTT092 | 2019/2/26 | 2019/4/7 | 2.6 | Nanjishan | Capture | Backpack (HQBG3621S) | *A. albifrons* |

**Table S2.** Model selection results for all-subsets multivariate Cox regression.

| Variables | AIC | LogLik | LR_Chisq | LR_pvalue | C_index | NumVars |
| --- | --- | --- | --- | --- | --- | --- |
| ActAvg+WDSP+Releasetime | 124.92 | -59.46 | 12.73 | 0.005 | 0.758 | 3 |
| ActAvg+WDSP | 125.07 | -60.54 | 10.58 | 0.005 | 0.726 | 2 |
| ActAvg+KDE+WDSP | 126.41 | -60.2 | 11.24 | 0.01 | 0.717 | 3 |
| ActAvg+KDE+WDSP+Releasetime | 126.55 | -59.28 | 13.1 | 0.011 | 0.732 | 4 |
| ActAvg+WDSP+PRCP | 126.81 | -60.41 | 10.84 | 0.013 | 0.723 | 3 |
| StayDur+ActAvg+WDSP+Releasetime | 126.88 | -59.44 | 12.77 | 0.012 | 0.76 | 4 |
| ActAvg+WDSP+PRCP+Releasetime | 126.9 | -59.45 | 12.75 | 0.013 | 0.749 | 4 |
| ActAvg+TEMP+WDSP+Releasetime | 126.91 | -59.46 | 12.74 | 0.013 | 0.757 | 4 |
| ActAvg+VISIB+WDSP+Releasetime | 126.91 | -59.46 | 12.74 | 0.013 | 0.751 | 4 |
| ActAvg+Weight+WDSP+Releasetime | 126.92 | -59.46 | 12.73 | 0.013 | 0.755 | 4 |
| ActAvg+TEMP+WDSP | 127.05 | -60.53 | 10.6 | 0.014 | 0.723 | 3 |
| StayDur+ActAvg+WDSP | 127.06 | -60.53 | 10.59 | 0.014 | 0.728 | 3 |
| ActAvg+VISIB+WDSP | 127.06 | -60.53 | 10.59 | 0.014 | 0.734 | 3 |
| ActAvg+Weight+WDSP | 127.07 | -60.54 | 10.58 | 0.014 | 0.728 | 3 |
| ActAvg+KDE+WDSP+PRCP | 128.15 | -60.07 | 11.5 | 0.021 | 0.721 | 4 |
| StayDur+ActAvg+KDE+WDSP | 128.28 | -60.14 | 11.37 | 0.023 | 0.719 | 4 |
| ActAvg+KDE+VISIB+WDSP | 128.33 | -60.16 | 11.32 | 0.023 | 0.719 | 4 |
| ActAvg+KDE+Weight+WDSP | 128.34 | -60.17 | 11.31 | 0.023 | 0.717 | 4 |
| StayDur+ActAvg+KDE+WDSP+Releasetime | 128.39 | -59.2 | 13.26 | 0.021 | 0.74 | 5 |
| ActAvg+KDE+TEMP+WDSP | 128.41 | -60.2 | 11.24 | 0.024 | 0.717 | 4 |
| ActAvg+KDE+Weight+WDSP+Releasetime | 128.47 | -59.24 | 13.18 | 0.022 | 0.725 | 5 |
| ActAvg+KDE+WDSP+PRCP+Releasetime | 128.51 | -59.26 | 13.14 | 0.022 | 0.74 | 5 |
| ActAvg+KDE+TEMP+WDSP+Releasetime | 128.55 | -59.28 | 13.1 | 0.022 | 0.732 | 5 |
| ActAvg+KDE+VISIB+WDSP+Releasetime | 128.55 | -59.27 | 13.1 | 0.022 | 0.738 | 5 |
| ActAvg+VISIB+WDSP+PRCP | 128.73 | -60.36 | 10.93 | 0.027 | 0.726 | 4 |
| ActAvg+Weight+WDSP+PRCP | 128.74 | -60.37 | 10.91 | 0.028 | 0.725 | 4 |
| ActAvg+TEMP+WDSP+PRCP | 128.79 | -60.4 | 10.86 | 0.028 | 0.725 | 4 |
| StayDur+ActAvg+WDSP+PRCP | 128.81 | -60.4 | 10.84 | 0.028 | 0.723 | 4 |
| StayDur+ActAvg+TEMP+WDSP+Releasetime | 128.83 | -59.41 | 12.82 | 0.025 | 0.749 | 5 |
| StayDur+ActAvg+VISIB+WDSP+Releasetime | 128.86 | -59.43 | 12.79 | 0.025 | 0.749 | 5 |
| StayDur+ActAvg+Weight+WDSP+Releasetime | 128.88 | -59.44 | 12.77 | 0.026 | 0.76 | 5 |
| StayDur+ActAvg+WDSP+PRCP+Releasetime | 128.88 | -59.44 | 12.78 | 0.026 | 0.76 | 5 |
| WDSP+Releasetime | 128.9 | -62.45 | 6.75 | 0.034 | 0.67 | 2 |
| ActAvg+Weight+WDSP+PRCP+Releasetime | 128.9 | -59.45 | 12.75 | 0.026 | 0.749 | 5 |
| ActAvg+TEMP+WDSP+PRCP+Releasetime | 128.9 | -59.45 | 12.76 | 0.026 | 0.743 | 5 |
| ActAvg+VISIB+WDSP+PRCP+Releasetime | 128.9 | -59.45 | 12.75 | 0.026 | 0.751 | 5 |
| ActAvg+Weight+TEMP+WDSP+Releasetime | 128.91 | -59.45 | 12.74 | 0.026 | 0.751 | 5 |
| ActAvg+Weight+VISIB+WDSP+Releasetime | 128.91 | -59.46 | 12.74 | 0.026 | 0.749 | 5 |
| ActAvg+TEMP+VISIB+WDSP+Releasetime | 128.91 | -59.46 | 12.74 | 0.026 | 0.753 | 5 |
| StayDur+ActAvg+TEMP+WDSP | 129 | -60.5 | 10.65 | 0.031 | 0.721 | 4 |
| ActAvg+TEMP+VISIB+WDSP | 129.01 | -60.5 | 10.64 | 0.031 | 0.721 | 4 |
| StayDur+ActAvg+VISIB+WDSP | 129.05 | -60.53 | 10.6 | 0.031 | 0.73 | 4 |
| ActAvg+Weight+TEMP+WDSP | 129.05 | -60.53 | 10.6 | 0.031 | 0.723 | 4 |
| StayDur+ActAvg+Weight+WDSP | 129.06 | -60.53 | 10.6 | 0.032 | 0.728 | 4 |
| ActAvg+Weight+VISIB+WDSP | 129.06 | -60.53 | 10.59 | 0.032 | 0.734 | 4 |
| StayDur+Releasetime | 129.77 | -62.89 | 5.88 | 0.053 | 0.686 | 2 |
| StayDur+WDSP+Releasetime | 129.89 | -61.95 | 7.76 | 0.051 | 0.718 | 3 |
| ActAvg+KDE+VISIB+WDSP+PRCP | 129.89 | -59.95 | 11.76 | 0.038 | 0.723 | 5 |
| ActAvg+TEMP | 129.96 | -62.98 | 5.69 | 0.058 | 0.653 | 2 |
| StayDur+ActAvg+Releasetime | 129.97 | -61.99 | 7.68 | 0.053 | 0.687 | 3 |
| StayDur+ActAvg+KDE+WDSP+PRCP | 130.11 | -60.05 | 11.54 | 0.042 | 0.728 | 5 |
| ActAvg+KDE+Weight+WDSP+PRCP | 130.15 | -60.07 | 11.51 | 0.042 | 0.721 | 5 |
| ActAvg+KDE+TEMP+WDSP+PRCP | 130.15 | -60.07 | 11.5 | 0.042 | 0.719 | 5 |
| StayDur+ActAvg+KDE+VISIB+WDSP | 130.21 | -60.11 | 11.44 | 0.043 | 0.717 | 5 |
| ActAvg+KDE+Weight+VISIB+WDSP | 130.23 | -60.12 | 11.42 | 0.044 | 0.725 | 5 |
| StayDur+ActAvg+KDE+Weight+WDSP | 130.24 | -60.12 | 11.41 | 0.044 | 0.719 | 5 |
| StayDur+ActAvg+KDE+TEMP+WDSP | 130.24 | -60.12 | 11.41 | 0.044 | 0.719 | 5 |
| ActAvg+Releasetime | 130.25 | -63.12 | 5.41 | 0.067 | 0.677 | 2 |
| StayDur+ActAvg | 130.26 | -63.13 | 5.39 | 0.067 | 0.643 | 2 |
| ActAvg+KDE+TEMP+VISIB+WDSP | 130.29 | -60.15 | 11.36 | 0.045 | 0.717 | 5 |
| ActAvg+TEMP+Releasetime | 130.3 | -62.15 | 7.35 | 0.061 | 0.683 | 3 |
| ActAvg+KDE+Weight+TEMP+WDSP | 130.33 | -60.17 | 11.32 | 0.045 | 0.721 | 5 |
| StayDur+ActAvg+KDE+Weight+WDSP+Releasetime | 130.35 | -59.18 | 13.3 | 0.039 | 0.728 | 6 |
| StayDur+ActAvg+KDE+TEMP+WDSP+Releasetime | 130.36 | -59.18 | 13.29 | 0.039 | 0.734 | 6 |
| StayDur+ActAvg+KDE+VISIB+WDSP+Releasetime | 130.39 | -59.2 | 13.26 | 0.039 | 0.74 | 6 |
| StayDur+ActAvg+KDE+WDSP+PRCP+Releasetime | 130.39 | -59.2 | 13.26 | 0.039 | 0.74 | 6 |
| ActAvg+KDE+Weight+VISIB+WDSP+Releasetime | 130.46 | -59.23 | 13.19 | 0.04 | 0.728 | 6 |
| ActAvg+KDE+Weight+TEMP+WDSP+Releasetime | 130.47 | -59.24 | 13.18 | 0.04 | 0.725 | 6 |
| ActAvg+KDE+Weight+WDSP+PRCP+Releasetime | 130.47 | -59.23 | 13.18 | 0.04 | 0.725 | 6 |
| WDSP+PRCP+Releasetime | 130.48 | -62.24 | 7.17 | 0.067 | 0.675 | 3 |
| ActAvg+KDE+VISIB+WDSP+PRCP+Releasetime | 130.49 | -59.25 | 13.16 | 0.041 | 0.728 | 6 |
| ActAvg+KDE+TEMP+WDSP+PRCP+Releasetime | 130.51 | -59.26 | 13.14 | 0.041 | 0.736 | 6 |
| Weight+WDSP+Releasetime | 130.54 | -62.27 | 7.11 | 0.068 | 0.682 | 3 |
| ActAvg+KDE+TEMP+VISIB+WDSP+Releasetime | 130.55 | -59.27 | 13.1 | 0.041 | 0.738 | 6 |
| ActAvg+TEMP+VISIB+WDSP+PRCP | 130.57 | -60.28 | 11.08 | 0.05 | 0.723 | 5 |
| TEMP+Releasetime | 130.6 | -63.3 | 5.05 | 0.08 | 0.661 | 2 |
| ActAvg+Weight+VISIB+WDSP+PRCP | 130.63 | -60.32 | 11.02 | 0.051 | 0.732 | 5 |
| TEMP+WDSP+Releasetime | 130.64 | -62.32 | 7.01 | 0.071 | 0.697 | 3 |
| StayDur | 130.67 | -64.33 | 2.99 | 0.084 | 0.667 | 1 |
| Releasetime | 130.71 | -64.35 | 2.94 | 0.086 | 0.6 | 1 |
| StayDur+ActAvg+VISIB+WDSP+PRCP | 130.72 | -60.36 | 10.94 | 0.053 | 0.73 | 5 |
| StayDur+ActAvg+Weight+WDSP+PRCP | 130.74 | -60.37 | 10.91 | 0.053 | 0.725 | 5 |
| ActAvg+Weight+TEMP+WDSP+PRCP | 130.74 | -60.37 | 10.92 | 0.053 | 0.726 | 5 |
| WDSP | 130.76 | -64.38 | 2.89 | 0.089 | 0.615 | 1 |
| StayDur+ActAvg+TEMP+WDSP+PRCP | 130.79 | -60.39 | 10.86 | 0.054 | 0.725 | 5 |
| StayDur+ActAvg+Weight+TEMP+WDSP+Releasetime | 130.83 | -59.41 | 12.83 | 0.046 | 0.749 | 6 |
| StayDur+ActAvg+TEMP+VISIB+WDSP+Releasetime | 130.83 | -59.41 | 12.82 | 0.046 | 0.749 | 6 |
| StayDur+ActAvg+TEMP+WDSP+PRCP+Releasetime | 130.83 | -59.41 | 12.82 | 0.046 | 0.749 | 6 |
| StayDur+ActAvg+Weight+VISIB+WDSP+Releasetime | 130.86 | -59.43 | 12.79 | 0.047 | 0.753 | 6 |
| StayDur+ActAvg+VISIB+WDSP+PRCP+Releasetime | 130.86 | -59.43 | 12.79 | 0.047 | 0.751 | 6 |
| ActAvg | 130.87 | -64.43 | 2.78 | 0.095 | 0.613 | 1 |
| StayDur+ActAvg+Weight+WDSP+PRCP+Releasetime | 130.87 | -59.44 | 12.78 | 0.047 | 0.76 | 6 |
| KDE+WDSP+Releasetime | 130.88 | -62.44 | 6.77 | 0.079 | 0.664 | 3 |
| VISIB+WDSP+Releasetime | 130.9 | -62.45 | 6.75 | 0.08 | 0.673 | 3 |
| ActAvg+Weight+TEMP+WDSP+PRCP+Releasetime | 130.9 | -59.45 | 12.76 | 0.047 | 0.745 | 6 |
| ActAvg+Weight+VISIB+WDSP+PRCP+Releasetime | 130.9 | -59.45 | 12.75 | 0.047 | 0.749 | 6 |
| ActAvg+TEMP+VISIB+WDSP+PRCP+Releasetime | 130.9 | -59.45 | 12.76 | 0.047 | 0.745 | 6 |
| ActAvg+Weight+TEMP+VISIB+WDSP+Releasetime | 130.91 | -59.45 | 12.74 | 0.047 | 0.751 | 6 |
| StayDur+ActAvg+TEMP+VISIB+WDSP | 130.93 | -60.46 | 10.72 | 0.057 | 0.719 | 5 |
| StayDur+ActAvg+Weight+TEMP+WDSP | 131 | -60.5 | 10.65 | 0.059 | 0.723 | 5 |
| ActAvg+Weight+TEMP+VISIB+WDSP | 131 | -60.5 | 10.65 | 0.059 | 0.721 | 5 |
| TEMP | 131.02 | -64.51 | 2.63 | 0.105 | 0.626 | 1 |
| StayDur+ActAvg+Weight+VISIB+WDSP | 131.05 | -60.52 | 10.6 | 0.06 | 0.734 | 5 |
| ActAvg+TEMP+VISIB+Releasetime | 131.16 | -61.58 | 8.49 | 0.075 | 0.704 | 4 |
| StayDur+ActAvg+KDE | 131.22 | -62.61 | 6.43 | 0.092 | 0.668 | 3 |
| StayDur+ActAvg+KDE+Releasetime | 131.26 | -61.63 | 8.39 | 0.078 | 0.681 | 4 |
| ActAvg+KDE+TEMP | 131.3 | -62.65 | 6.35 | 0.096 | 0.67 | 3 |
| StayDur+KDE+Releasetime | 131.33 | -62.67 | 6.32 | 0.097 | 0.668 | 3 |
| ActAvg+TEMP+VISIB | 131.36 | -62.68 | 6.29 | 0.098 | 0.655 | 3 |
| WDSP+PRCP | 131.38 | -63.69 | 4.27 | 0.118 | 0.666 | 2 |
| ActAvg+TEMP+PRCP | 131.42 | -62.71 | 6.23 | 0.101 | 0.658 | 3 |
| StayDur+TEMP+Releasetime | 131.54 | -62.77 | 6.11 | 0.106 | 0.69 | 3 |
| StayDur+ActAvg+TEMP | 131.55 | -62.78 | 6.1 | 0.107 | 0.653 | 3 |
| ActAvg+PRCP | 131.6 | -63.8 | 4.05 | 0.132 | 0.647 | 2 |
| StayDur+KDE+WDSP+Releasetime | 131.62 | -61.81 | 8.03 | 0.09 | 0.685 | 4 |
| StayDur+WDSP | 131.63 | -63.82 | 4.02 | 0.134 | 0.656 | 2 |
| StayDur+Weight+Releasetime | 131.65 | -62.83 | 6 | 0.112 | 0.684 | 3 |
| StayDur+VISIB+Releasetime | 131.66 | -62.83 | 5.99 | 0.112 | 0.673 | 3 |
| StayDur+ActAvg+TEMP+Releasetime | 131.66 | -61.83 | 7.99 | 0.092 | 0.692 | 4 |
| StayDur+ActAvg+VISIB+Releasetime | 131.71 | -61.86 | 7.94 | 0.094 | 0.683 | 4 |
| TEMP+PRCP | 131.76 | -63.88 | 3.89 | 0.143 | 0.63 | 2 |
| ActAvg+KDE+TEMP+VISIB+WDSP+PRCP | 131.76 | -59.88 | 11.89 | 0.064 | 0.719 | 6 |
| StayDur+PRCP+Releasetime | 131.77 | -62.89 | 5.88 | 0.118 | 0.684 | 3 |
| StayDur+Weight+WDSP+Releasetime | 131.77 | -61.88 | 7.88 | 0.096 | 0.714 | 4 |
| StayDur+WDSP+PRCP+Releasetime | 131.79 | -61.9 | 7.86 | 0.097 | 0.697 | 4 |
| StayDur+KDE | 131.81 | -63.91 | 3.84 | 0.147 | 0.643 | 2 |
| TEMP+VISIB+Releasetime | 131.84 | -62.92 | 5.81 | 0.121 | 0.678 | 3 |
| ActAvg+KDE+TEMP+Releasetime | 131.86 | -61.93 | 7.79 | 0.1 | 0.683 | 4 |
| StayDur+VISIB+WDSP+Releasetime | 131.88 | -61.94 | 7.77 | 0.1 | 0.703 | 4 |
| StayDur+TEMP+WDSP+Releasetime | 131.89 | -61.95 | 7.76 | 0.101 | 0.71 | 4 |
| StayDur+ActAvg+KDE+VISIB+WDSP+PRCP | 131.89 | -59.94 | 11.77 | 0.067 | 0.721 | 6 |
| ActAvg+KDE+Weight+VISIB+WDSP+PRCP | 131.89 | -59.95 | 11.76 | 0.068 | 0.723 | 6 |
| ActAvg+Weight+TEMP | 131.91 | -62.96 | 5.74 | 0.125 | 0.651 | 3 |
| StayDur+ActAvg+Weight+Releasetime | 131.93 | -61.97 | 7.72 | 0.103 | 0.674 | 4 |
| StayDur+ActAvg+PRCP+Releasetime | 131.95 | -61.98 | 7.7 | 0.103 | 0.689 | 4 |
| ActAvg+Weight+Releasetime | 131.96 | -62.98 | 5.69 | 0.128 | 0.677 | 3 |
| ActAvg+VISIB+Releasetime | 131.96 | -62.98 | 5.69 | 0.128 | 0.66 | 3 |
| StayDur+ActAvg+PRCP | 131.98 | -62.99 | 5.67 | 0.129 | 0.655 | 3 |
| ActAvg+PRCP+Releasetime | 132.01 | -63 | 5.65 | 0.13 | 0.662 | 3 |
| ActAvg+KDE+Releasetime | 132.03 | -63.02 | 5.62 | 0.132 | 0.681 | 3 |
| StayDur+ActAvg+KDE+TEMP+VISIB+WDSP | 132.03 | -60.02 | 11.62 | 0.071 | 0.719 | 6 |
| StayDur+PRCP | 132.07 | -64.03 | 3.58 | 0.167 | 0.652 | 2 |
| StayDur+ActAvg+KDE+TEMP+WDSP+PRCP | 132.1 | -60.05 | 11.55 | 0.073 | 0.726 | 6 |
| StayDur+ActAvg+KDE+Weight+WDSP+PRCP | 132.11 | -60.05 | 11.54 | 0.073 | 0.721 | 6 |
| ActAvg+KDE+Weight+TEMP+VISIB+WDSP | 132.14 | -60.07 | 11.51 | 0.074 | 0.721 | 6 |
| StayDur+ActAvg+VISIB | 132.15 | -63.07 | 5.5 | 0.138 | 0.638 | 3 |
| StayDur+ActAvg+KDE+Weight+VISIB+WDSP | 132.15 | -60.08 | 11.5 | 0.074 | 0.711 | 6 |
| ActAvg+KDE+Weight+TEMP+WDSP+PRCP | 132.15 | -60.07 | 11.51 | 0.074 | 0.719 | 6 |
| PRCP | 132.17 | -65.09 | 1.48 | 0.224 | 0.609 | 1 |
| StayDur+ActAvg+KDE+Weight+TEMP+WDSP | 132.19 | -60.09 | 11.46 | 0.075 | 0.725 | 6 |
| ActAvg+TEMP+PRCP+Releasetime | 132.2 | -62.1 | 7.45 | 0.114 | 0.689 | 4 |
| TEMP+WDSP+PRCP+Releasetime | 132.2 | -62.1 | 7.45 | 0.114 | 0.705 | 4 |
| Weight+Releasetime | 132.22 | -64.11 | 3.43 | 0.18 | 0.629 | 2 |
| StayDur+ActAvg+Weight | 132.22 | -63.11 | 5.43 | 0.143 | 0.645 | 3 |
| ActAvg+Weight+TEMP+Releasetime | 132.24 | -62.12 | 7.41 | 0.116 | 0.675 | 4 |
| StayDur+TEMP | 132.25 | -64.12 | 3.4 | 0.182 | 0.646 | 2 |
| TEMP+PRCP+Releasetime | 132.26 | -63.13 | 5.39 | 0.145 | 0.665 | 3 |
| TEMP+WDSP | 132.27 | -64.13 | 3.38 | 0.184 | 0.647 | 2 |
| StayDur+ActAvg+KDE+Weight+TEMP+WDSP+Releasetime | 132.3 | -59.15 | 13.35 | 0.064 | 0.734 | 7 |
| PRCP+Releasetime | 132.33 | -64.17 | 3.32 | 0.19 | 0.606 | 2 |
| StayDur+ActAvg+KDE+TEMP+VISIB+WDSP+Releasetime | 132.33 | -59.17 | 13.32 | 0.065 | 0.736 | 7 |
| Weight+WDSP+PRCP+Releasetime | 132.35 | -62.17 | 7.3 | 0.121 | 0.671 | 4 |
| StayDur+ActAvg+KDE+Weight+VISIB+WDSP+Releasetime | 132.35 | -59.17 | 13.3 | 0.065 | 0.728 | 7 |
| StayDur+ActAvg+KDE+Weight+WDSP+PRCP+Releasetime | 132.35 | -59.17 | 13.3 | 0.065 | 0.726 | 7 |
| StayDur+ActAvg+KDE+TEMP+WDSP+PRCP+Releasetime | 132.36 | -59.18 | 13.29 | 0.065 | 0.736 | 7 |
| KDE+WDSP | 132.37 | -64.19 | 3.28 | 0.194 | 0.594 | 2 |
| StayDur+ActAvg+KDE+VISIB+WDSP+PRCP+Releasetime | 132.39 | -59.2 | 13.26 | 0.066 | 0.738 | 7 |
| ActAvg+KDE | 132.4 | -64.2 | 3.25 | 0.196 | 0.613 | 2 |
| Weight+TEMP+WDSP+Releasetime | 132.42 | -62.21 | 7.23 | 0.124 | 0.697 | 4 |
| VISIB+WDSP+PRCP+Releasetime | 132.42 | -62.21 | 7.23 | 0.124 | 0.69 | 4 |
| KDE+WDSP+PRCP+Releasetime | 132.43 | -62.22 | 7.22 | 0.125 | 0.668 | 4 |
| Weight+WDSP | 132.44 | -64.22 | 3.21 | 0.201 | 0.641 | 2 |
| KDE+TEMP+Releasetime | 132.45 | -63.23 | 5.2 | 0.158 | 0.658 | 3 |
| ActAvg+KDE+Weight+TEMP+VISIB+WDSP+Releasetime | 132.45 | -59.22 | 13.2 | 0.067 | 0.723 | 7 |
| ActAvg+KDE+Weight+VISIB+WDSP+PRCP+Releasetime | 132.45 | -59.23 | 13.2 | 0.067 | 0.719 | 7 |
| Weight+TEMP+Releasetime | 132.47 | -63.23 | 5.19 | 0.159 | 0.656 | 3 |
| KDE+Weight+WDSP+Releasetime | 132.47 | -62.23 | 7.18 | 0.126 | 0.67 | 4 |
| ActAvg+KDE+Weight+TEMP+WDSP+PRCP+Releasetime | 132.47 | -59.23 | 13.18 | 0.068 | 0.723 | 7 |
| ActAvg+KDE+TEMP+VISIB+WDSP+PRCP+Releasetime | 132.49 | -59.24 | 13.17 | 0.068 | 0.736 | 7 |
| KDE+TEMP | 132.52 | -64.26 | 3.13 | 0.209 | 0.611 | 2 |
| TEMP+VISIB+WDSP+Releasetime | 132.52 | -62.26 | 7.13 | 0.129 | 0.707 | 4 |
| ActAvg+Weight+TEMP+VISIB+WDSP+PRCP | 132.52 | -60.26 | 11.13 | 0.084 | 0.732 | 6 |
| Weight+VISIB+WDSP+Releasetime | 132.53 | -62.26 | 7.12 | 0.13 | 0.684 | 4 |
| StayDur+Weight | 132.54 | -64.27 | 3.11 | 0.211 | 0.656 | 2 |
| StayDur+ActAvg+KDE+TEMP | 132.54 | -62.27 | 7.11 | 0.13 | 0.668 | 4 |
| VISIB+Releasetime | 132.55 | -64.27 | 3.1 | 0.212 | 0.571 | 2 |
| StayDur+ActAvg+TEMP+VISIB+WDSP+PRCP | 132.56 | -60.28 | 11.09 | 0.085 | 0.725 | 6 |
| KDE+TEMP+WDSP+Releasetime | 132.57 | -62.28 | 7.09 | 0.131 | 0.692 | 4 |
| ActAvg+VISIB | 132.63 | -64.32 | 3.02 | 0.221 | 0.615 | 2 |
| StayDur+ActAvg+Weight+VISIB+WDSP+PRCP | 132.63 | -60.31 | 11.02 | 0.088 | 0.736 | 6 |
| ActAvg+Weight | 132.64 | -64.32 | 3.01 | 0.222 | 0.619 | 2 |
| StayDur+VISIB | 132.66 | -64.33 | 2.99 | 0.224 | 0.665 | 2 |
| KDE+Releasetime | 132.7 | -64.35 | 2.95 | 0.228 | 0.56 | 2 |
| VISIB+WDSP | 132.72 | -64.36 | 2.93 | 0.231 | 0.619 | 2 |
| StayDur+WDSP+PRCP | 132.73 | -63.36 | 4.92 | 0.178 | 0.663 | 3 |
| StayDur+ActAvg+Weight+TEMP+WDSP+PRCP | 132.73 | -60.37 | 10.92 | 0.091 | 0.723 | 6 |
| TEMP+VISIB | 132.81 | -64.4 | 2.84 | 0.242 | 0.645 | 2 |
| TEMP+WDSP+PRCP | 132.82 | -63.41 | 4.84 | 0.184 | 0.67 | 3 |
| StayDur+ActAvg+Weight+TEMP+VISIB+WDSP+Releasetime | 132.82 | -59.41 | 12.83 | 0.076 | 0.751 | 7 |
| StayDur+KDE+WDSP | 132.83 | -63.41 | 4.82 | 0.185 | 0.657 | 3 |
| StayDur+ActAvg+Weight+TEMP+WDSP+PRCP+Releasetime | 132.83 | -59.41 | 12.83 | 0.076 | 0.749 | 7 |
| StayDur+ActAvg+TEMP+VISIB+WDSP+PRCP+Releasetime | 132.83 | -59.41 | 12.83 | 0.076 | 0.749 | 7 |
| Weight+TEMP | 132.86 | -64.43 | 2.79 | 0.248 | 0.629 | 2 |
| StayDur+ActAvg+TEMP+VISIB+Releasetime | 132.86 | -61.43 | 8.79 | 0.118 | 0.711 | 5 |
| StayDur+ActAvg+Weight+VISIB+WDSP+PRCP+Releasetime | 132.86 | -59.43 | 12.79 | 0.077 | 0.753 | 7 |
| KDE+VISIB+WDSP+Releasetime | 132.88 | -62.44 | 6.77 | 0.148 | 0.664 | 4 |
| ActAvg+Weight+TEMP+VISIB+WDSP+PRCP+Releasetime | 132.9 | -59.45 | 12.76 | 0.078 | 0.743 | 7 |
| ActAvg+KDE+TEMP+PRCP | 132.91 | -62.46 | 6.74 | 0.15 | 0.666 | 4 |
| ActAvg+KDE+TEMP+VISIB | 132.92 | -62.46 | 6.73 | 0.151 | 0.67 | 4 |
| StayDur+ActAvg+KDE+TEMP+Releasetime | 132.92 | -61.46 | 8.73 | 0.12 | 0.696 | 5 |
| StayDur+ActAvg+Weight+TEMP+VISIB+WDSP | 132.93 | -60.46 | 10.72 | 0.097 | 0.719 | 6 |
| ActAvg+KDE+TEMP+VISIB+Releasetime | 132.97 | -61.49 | 8.68 | 0.123 | 0.704 | 5 |
| VISIB+WDSP+PRCP | 132.99 | -63.49 | 4.66 | 0.198 | 0.681 | 3 |
| StayDur+KDE+TEMP+Releasetime | 133.06 | -62.53 | 6.59 | 0.159 | 0.683 | 4 |
| StayDur+ActAvg+KDE+Weight | 133.07 | -62.53 | 6.58 | 0.16 | 0.662 | 4 |
| StayDur+ActAvg+TEMP+VISIB | 133.12 | -62.56 | 6.53 | 0.163 | 0.664 | 4 |
| ActAvg+TEMP+VISIB+PRCP+Releasetime | 133.12 | -61.56 | 8.54 | 0.129 | 0.713 | 5 |
| KDE+WDSP+PRCP | 133.13 | -63.57 | 4.52 | 0.21 | 0.651 | 3 |
| StayDur+TEMP+VISIB+Releasetime | 133.13 | -62.57 | 6.52 | 0.164 | 0.697 | 4 |
| ActAvg+TEMP+VISIB+PRCP | 133.13 | -62.56 | 6.52 | 0.163 | 0.658 | 4 |
| ActAvg+Weight+TEMP+VISIB+Releasetime | 133.14 | -61.57 | 8.51 | 0.13 | 0.711 | 5 |
| StayDur+KDE+Weight+Releasetime | 133.15 | -62.57 | 6.5 | 0.165 | 0.672 | 4 |
| StayDur+ActAvg+KDE+Weight+Releasetime | 133.16 | -61.58 | 8.49 | 0.131 | 0.677 | 5 |
| StayDur+ActAvg+KDE+PRCP+Releasetime | 133.16 | -61.58 | 8.49 | 0.131 | 0.683 | 5 |
| StayDur+ActAvg+KDE+PRCP | 133.17 | -62.59 | 6.48 | 0.166 | 0.664 | 4 |
| ActAvg+KDE+Weight+TEMP | 133.17 | -62.58 | 6.48 | 0.166 | 0.67 | 4 |
| StayDur+ActAvg+KDE+VISIB+Releasetime | 133.19 | -61.6 | 8.46 | 0.133 | 0.675 | 5 |
| StayDur+ActAvg+KDE+VISIB | 133.22 | -62.61 | 6.43 | 0.169 | 0.67 | 4 |
| StayDur+ActAvg+TEMP+PRCP | 133.23 | -62.61 | 6.42 | 0.17 | 0.666 | 4 |
| Weight | 133.25 | -65.62 | 0.4 | 0.526 | 0.523 | 1 |
| Weight+WDSP+PRCP | 133.3 | -63.65 | 4.35 | 0.226 | 0.665 | 3 |
| StayDur+KDE+VISIB+Releasetime | 133.31 | -62.66 | 6.34 | 0.175 | 0.668 | 4 |
| StayDur+KDE+PRCP+Releasetime | 133.33 | -62.66 | 6.32 | 0.176 | 0.668 | 4 |
| StayDur+KDE+TEMP | 133.34 | -63.67 | 4.31 | 0.23 | 0.657 | 3 |
| ActAvg+Weight+TEMP+VISIB | 133.36 | -62.68 | 6.29 | 0.178 | 0.657 | 4 |
| ActAvg+KDE+PRCP | 133.4 | -63.7 | 4.25 | 0.235 | 0.634 | 3 |
| KDE | 133.41 | -65.71 | 0.24 | 0.624 | 0.474 | 1 |
| KDE+TEMP+PRCP | 133.41 | -63.71 | 4.24 | 0.237 | 0.623 | 3 |
| ActAvg+Weight+TEMP+PRCP | 133.42 | -62.71 | 6.23 | 0.183 | 0.66 | 4 |
| StayDur+TEMP+PRCP | 133.43 | -63.72 | 4.22 | 0.239 | 0.654 | 3 |
| StayDur+KDE+Weight+WDSP+Releasetime | 133.43 | -61.72 | 8.22 | 0.144 | 0.681 | 5 |
| StayDur+Weight+TEMP+Releasetime | 133.46 | -62.73 | 6.19 | 0.186 | 0.678 | 4 |
| StayDur+ActAvg+VISIB+PRCP+Releasetime | 133.48 | -61.74 | 8.17 | 0.147 | 0.708 | 5 |
| StayDur+Weight+WDSP | 133.51 | -63.76 | 4.14 | 0.247 | 0.686 | 3 |
| StayDur+TEMP+PRCP+Releasetime | 133.51 | -62.75 | 6.14 | 0.189 | 0.688 | 4 |
| StayDur+KDE+PRCP | 133.53 | -63.77 | 4.12 | 0.249 | 0.64 | 3 |
| ActAvg+Weight+PRCP | 133.54 | -63.77 | 4.11 | 0.249 | 0.658 | 3 |
| StayDur+ActAvg+Weight+TEMP | 133.54 | -62.77 | 6.12 | 0.191 | 0.658 | 4 |
| StayDur+KDE+WDSP+PRCP+Releasetime | 133.54 | -61.77 | 8.12 | 0.15 | 0.683 | 5 |
| StayDur+KDE+Weight | 133.56 | -63.78 | 4.09 | 0.252 | 0.638 | 3 |
| StayDur+Weight+VISIB+Releasetime | 133.57 | -62.79 | 6.08 | 0.193 | 0.682 | 4 |
| VISIB | 133.58 | -65.79 | 0.07 | 0.795 | 0.492 | 1 |
| ActAvg+VISIB+PRCP | 133.6 | -63.8 | 4.05 | 0.256 | 0.653 | 3 |
| StayDur+TEMP+WDSP | 133.61 | -63.8 | 4.04 | 0.257 | 0.663 | 3 |
| StayDur+VISIB+WDSP | 133.62 | -63.81 | 4.03 | 0.258 | 0.661 | 3 |
| StayDur+KDE+TEMP+WDSP+Releasetime | 133.62 | -61.81 | 8.03 | 0.155 | 0.687 | 5 |
| StayDur+KDE+VISIB+WDSP+Releasetime | 133.62 | -61.81 | 8.03 | 0.155 | 0.687 | 5 |
| StayDur+ActAvg+Weight+TEMP+Releasetime | 133.64 | -61.82 | 8.01 | 0.156 | 0.691 | 5 |
| StayDur+Weight+PRCP+Releasetime | 133.65 | -62.82 | 6 | 0.199 | 0.68 | 4 |
| StayDur+VISIB+PRCP+Releasetime | 133.65 | -62.82 | 6 | 0.199 | 0.676 | 4 |
| ActAvg+KDE+Weight+Releasetime | 133.66 | -62.83 | 5.99 | 0.2 | 0.66 | 4 |
| StayDur+ActAvg+TEMP+PRCP+Releasetime | 133.66 | -61.83 | 7.99 | 0.157 | 0.692 | 5 |
| StayDur+ActAvg+KDE+TEMP+VISIB+WDSP+PRCP | 133.66 | -59.83 | 11.99 | 0.101 | 0.719 | 7 |
| StayDur+ActAvg+Weight+VISIB+Releasetime | 133.7 | -61.85 | 7.96 | 0.159 | 0.681 | 5 |
| TEMP+VISIB+PRCP | 133.72 | -63.86 | 3.94 | 0.268 | 0.647 | 3 |
| KDE+TEMP+WDSP | 133.73 | -63.87 | 3.92 | 0.271 | 0.626 | 3 |
| StayDur+Weight+WDSP+PRCP+Releasetime | 133.73 | -61.87 | 7.92 | 0.161 | 0.703 | 5 |
| ActAvg+KDE+Weight+TEMP+VISIB+WDSP+PRCP | 133.74 | -59.87 | 11.91 | 0.104 | 0.723 | 7 |
| TEMP+VISIB+PRCP+Releasetime | 133.75 | -62.87 | 5.9 | 0.206 | 0.69 | 4 |
| Weight+TEMP+PRCP | 133.76 | -63.88 | 3.89 | 0.273 | 0.624 | 3 |
| StayDur+Weight+TEMP+WDSP+Releasetime | 133.76 | -61.88 | 7.89 | 0.162 | 0.701 | 5 |
| ActAvg+KDE+Weight+TEMP+Releasetime | 133.76 | -61.88 | 7.89 | 0.162 | 0.683 | 5 |
| StayDur+KDE+VISIB | 133.77 | -63.89 | 3.88 | 0.275 | 0.655 | 3 |
| StayDur+Weight+VISIB+WDSP+Releasetime | 133.77 | -61.88 | 7.88 | 0.163 | 0.708 | 5 |
| ActAvg+Weight+VISIB+Releasetime | 133.78 | -62.89 | 5.87 | 0.209 | 0.675 | 4 |
| StayDur+TEMP+WDSP+PRCP+Releasetime | 133.79 | -61.9 | 7.86 | 0.164 | 0.705 | 5 |
| StayDur+VISIB+WDSP+PRCP+Releasetime | 133.79 | -61.9 | 7.86 | 0.164 | 0.707 | 5 |
| ActAvg+KDE+TEMP+PRCP+Releasetime | 133.79 | -61.89 | 7.86 | 0.164 | 0.694 | 5 |
| KDE+TEMP+VISIB+Releasetime | 133.8 | -62.9 | 5.85 | 0.21 | 0.677 | 4 |
| ActAvg+KDE+PRCP+Releasetime | 133.84 | -62.92 | 5.81 | 0.213 | 0.67 | 4 |
| Weight+TEMP+VISIB+Releasetime | 133.84 | -62.92 | 5.81 | 0.214 | 0.667 | 4 |
| ActAvg+KDE+VISIB+Releasetime | 133.85 | -62.92 | 5.8 | 0.214 | 0.66 | 4 |
| ActAvg+Weight+PRCP+Releasetime | 133.85 | -62.93 | 5.8 | 0.215 | 0.662 | 4 |
| StayDur+TEMP+VISIB+WDSP+Releasetime | 133.88 | -61.94 | 7.77 | 0.169 | 0.71 | 5 |
| StayDur+ActAvg+KDE+Weight+VISIB+WDSP+PRCP | 133.88 | -59.94 | 11.77 | 0.109 | 0.723 | 7 |
| KDE+Weight+WDSP | 133.89 | -63.95 | 3.76 | 0.289 | 0.643 | 3 |
| StayDur+ActAvg+Weight+PRCP+Releasetime | 133.89 | -61.94 | 7.76 | 0.17 | 0.683 | 5 |
| ActAvg+VISIB+PRCP+Releasetime | 133.9 | -62.95 | 5.76 | 0.218 | 0.668 | 4 |
| StayDur+ActAvg+KDE+Weight+TEMP+VISIB+WDSP | 133.9 | -59.95 | 11.75 | 0.109 | 0.725 | 7 |
| Weight+PRCP | 133.93 | -64.96 | 1.72 | 0.423 | 0.629 | 2 |
| StayDur+ActAvg+Weight+PRCP | 133.97 | -62.99 | 5.68 | 0.225 | 0.647 | 4 |
| StayDur+ActAvg+VISIB+PRCP | 133.97 | -62.98 | 5.69 | 0.224 | 0.657 | 4 |
| ActAvg+KDE+Weight | 134.01 | -64.01 | 3.64 | 0.303 | 0.634 | 3 |
| StayDur+Weight+PRCP | 134.02 | -64.01 | 3.63 | 0.304 | 0.656 | 3 |
| Weight+PRCP+Releasetime | 134.02 | -64.01 | 3.63 | 0.304 | 0.625 | 3 |
| StayDur+VISIB+PRCP | 134.04 | -64.02 | 3.61 | 0.306 | 0.659 | 3 |
| KDE+TEMP+WDSP+PRCP+Releasetime | 134.07 | -62.03 | 7.58 | 0.181 | 0.698 | 5 |
| Weight+TEMP+WDSP | 134.09 | -64.04 | 3.57 | 0.312 | 0.656 | 3 |
| KDE+TEMP+PRCP+Releasetime | 134.09 | -63.04 | 5.56 | 0.234 | 0.662 | 4 |
| StayDur+ActAvg+KDE+Weight+TEMP+WDSP+PRCP | 134.09 | -60.05 | 11.56 | 0.116 | 0.728 | 7 |
| VISIB+PRCP | 134.1 | -65.05 | 1.55 | 0.461 | 0.619 | 2 |
| KDE+PRCP | 134.11 | -65.06 | 1.54 | 0.464 | 0.585 | 2 |
| StayDur+ActAvg+Weight+VISIB | 134.12 | -63.06 | 5.53 | 0.237 | 0.649 | 4 |
| StayDur+Weight+TEMP | 134.16 | -64.08 | 3.49 | 0.322 | 0.656 | 3 |
| StayDur+TEMP+VISIB | 134.16 | -64.08 | 3.49 | 0.322 | 0.65 | 3 |
| Weight+VISIB+Releasetime | 134.17 | -64.09 | 3.48 | 0.323 | 0.614 | 3 |
| Weight+TEMP+WDSP+PRCP+Releasetime | 134.17 | -62.08 | 7.48 | 0.187 | 0.703 | 5 |
| TEMP+VISIB+WDSP+PRCP+Releasetime | 134.18 | -62.09 | 7.47 | 0.188 | 0.693 | 5 |
| KDE+Weight+Releasetime | 134.19 | -64.09 | 3.47 | 0.325 | 0.628 | 3 |
| ActAvg+Weight+TEMP+PRCP+Releasetime | 134.19 | -62.09 | 7.47 | 0.188 | 0.681 | 5 |
| StayDur+KDE+WDSP+PRCP | 134.21 | -63.11 | 5.44 | 0.245 | 0.66 | 4 |
| Weight+TEMP+PRCP+Releasetime | 134.23 | -63.12 | 5.42 | 0.247 | 0.656 | 4 |
| StayDur+ActAvg+KDE+Weight+TEMP+VISIB+WDSP+Releasetime | 134.23 | -59.11 | 13.42 | 0.098 | 0.728 | 8 |
| KDE+VISIB+WDSP | 134.25 | -64.13 | 3.4 | 0.334 | 0.613 | 3 |
| TEMP+VISIB+WDSP | 134.26 | -64.13 | 3.39 | 0.335 | 0.645 | 3 |
| KDE+Weight+WDSP+PRCP+Releasetime | 134.26 | -62.13 | 7.39 | 0.193 | 0.658 | 5 |
| StayDur+ActAvg+KDE+Weight+TEMP+WDSP+PRCP+Releasetime | 134.28 | -59.14 | 13.37 | 0.1 | 0.73 | 8 |
| Weight+VISIB+WDSP+PRCP+Releasetime | 134.29 | -62.14 | 7.36 | 0.195 | 0.693 | 5 |
| ActAvg+KDE+VISIB | 134.3 | -64.15 | 3.35 | 0.341 | 0.608 | 3 |
| KDE+Weight+TEMP | 134.3 | -64.15 | 3.35 | 0.341 | 0.613 | 3 |
| KDE+Weight+TEMP+Releasetime | 134.3 | -63.15 | 5.36 | 0.253 | 0.649 | 4 |
| VISIB+PRCP+Releasetime | 134.31 | -64.15 | 3.34 | 0.342 | 0.575 | 3 |
| KDE+Weight+TEMP+WDSP+Releasetime | 134.31 | -62.16 | 7.34 | 0.197 | 0.685 | 5 |
| KDE+PRCP+Releasetime | 134.32 | -64.16 | 3.33 | 0.344 | 0.6 | 3 |
| StayDur+ActAvg+KDE+TEMP+VISIB+WDSP+PRCP+Releasetime | 134.32 | -59.16 | 13.33 | 0.101 | 0.734 | 8 |
| KDE+VISIB+WDSP+PRCP+Releasetime | 134.34 | -62.17 | 7.31 | 0.198 | 0.679 | 5 |
| Weight+VISIB+WDSP | 134.35 | -64.18 | 3.3 | 0.348 | 0.635 | 3 |
| StayDur+ActAvg+KDE+Weight+VISIB+WDSP+PRCP+Releasetime | 134.35 | -59.17 | 13.3 | 0.102 | 0.726 | 8 |
| KDE+TEMP+WDSP+PRCP | 134.39 | -63.2 | 5.26 | 0.262 | 0.672 | 4 |
| KDE+TEMP+VISIB | 134.4 | -64.2 | 3.25 | 0.355 | 0.63 | 3 |
| StayDur+ActAvg+KDE+TEMP+VISIB | 134.4 | -62.2 | 7.25 | 0.203 | 0.66 | 5 |
| Weight+TEMP+VISIB+WDSP+Releasetime | 134.4 | -62.2 | 7.25 | 0.203 | 0.695 | 5 |
| KDE+Weight+VISIB+WDSP+Releasetime | 134.43 | -62.22 | 7.22 | 0.205 | 0.674 | 5 |
| ActAvg+KDE+Weight+TEMP+VISIB+WDSP+PRCP+Releasetime | 134.43 | -59.21 | 13.22 | 0.104 | 0.713 | 8 |
| StayDur+ActAvg+KDE+TEMP+PRCP | 134.44 | -62.22 | 7.21 | 0.206 | 0.668 | 5 |
| StayDur+ActAvg+KDE+Weight+TEMP | 134.46 | -62.23 | 7.2 | 0.207 | 0.666 | 5 |
| ActAvg+Weight+VISIB | 134.47 | -64.24 | 3.18 | 0.365 | 0.625 | 3 |
| KDE+TEMP+VISIB+WDSP+Releasetime | 134.47 | -62.23 | 7.18 | 0.207 | 0.689 | 5 |
| StayDur+ActAvg+KDE+TEMP+VISIB+Releasetime | 134.48 | -61.24 | 9.17 | 0.164 | 0.7 | 6 |
| StayDur+VISIB+WDSP+PRCP | 134.49 | -63.25 | 5.16 | 0.271 | 0.682 | 4 |
| StayDur+ActAvg+Weight+TEMP+VISIB+WDSP+PRCP | 134.51 | -60.25 | 11.15 | 0.132 | 0.73 | 7 |
| StayDur+Weight+VISIB | 134.54 | -64.27 | 3.11 | 0.375 | 0.652 | 3 |
| KDE+VISIB+WDSP+PRCP | 134.54 | -63.27 | 5.11 | 0.277 | 0.683 | 4 |
| KDE+VISIB+Releasetime | 134.55 | -64.27 | 3.1 | 0.376 | 0.574 | 3 |
| StayDur+KDE+Weight+WDSP | 134.58 | -63.29 | 5.07 | 0.28 | 0.677 | 4 |
| StayDur+TEMP+WDSP+PRCP | 134.61 | -63.3 | 5.04 | 0.283 | 0.671 | 4 |
| StayDur+ActAvg+TEMP+VISIB+PRCP+Releasetime | 134.63 | -61.31 | 9.02 | 0.172 | 0.717 | 6 |
| StayDur+KDE+VISIB+WDSP | 134.68 | -63.34 | 4.97 | 0.29 | 0.672 | 4 |
| StayDur+Weight+WDSP+PRCP | 134.71 | -63.36 | 4.94 | 0.294 | 0.673 | 4 |
| Weight+TEMP+VISIB | 134.73 | -64.36 | 2.92 | 0.404 | 0.629 | 3 |
| ActAvg+KDE+TEMP+VISIB+PRCP | 134.73 | -62.37 | 6.92 | 0.227 | 0.664 | 5 |
| TEMP+VISIB+WDSP+PRCP | 134.75 | -63.37 | 4.9 | 0.297 | 0.67 | 4 |
| StayDur+KDE+TEMP+WDSP | 134.77 | -63.38 | 4.88 | 0.299 | 0.66 | 4 |
| Weight+TEMP+WDSP+PRCP | 134.81 | -63.41 | 4.84 | 0.304 | 0.665 | 4 |
| StayDur+ActAvg+Weight+TEMP+VISIB+WDSP+PRCP+Releasetime | 134.82 | -59.41 | 12.83 | 0.118 | 0.751 | 8 |
| StayDur+KDE+TEMP+PRCP | 134.84 | -63.42 | 4.81 | 0.307 | 0.651 | 4 |
| StayDur+ActAvg+Weight+TEMP+VISIB+Releasetime | 134.85 | -61.42 | 8.8 | 0.185 | 0.715 | 6 |
| StayDur+KDE+TEMP+VISIB+Releasetime | 134.86 | -62.43 | 6.79 | 0.236 | 0.679 | 5 |
| StayDur+ActAvg+KDE+Weight+TEMP+Releasetime | 134.86 | -61.43 | 8.79 | 0.186 | 0.689 | 6 |
| StayDur+ActAvg+KDE+TEMP+PRCP+Releasetime | 134.88 | -61.44 | 8.77 | 0.187 | 0.694 | 6 |
| ActAvg+KDE+Weight+TEMP+VISIB | 134.89 | -62.44 | 6.76 | 0.239 | 0.683 | 5 |
| ActAvg+KDE+Weight+TEMP+PRCP | 134.89 | -62.45 | 6.76 | 0.239 | 0.67 | 5 |
| KDE+Weight | 134.9 | -65.45 | 0.75 | 0.686 | 0.528 | 2 |
| Weight+VISIB+WDSP+PRCP | 134.9 | -63.45 | 4.75 | 0.314 | 0.665 | 4 |
| StayDur+ActAvg+KDE+VISIB+PRCP+Releasetime | 134.9 | -61.45 | 8.75 | 0.188 | 0.691 | 6 |
| StayDur+ActAvg+KDE+Weight+PRCP+Releasetime | 134.93 | -61.47 | 8.72 | 0.19 | 0.689 | 6 |
| ActAvg+KDE+TEMP+VISIB+PRCP+Releasetime | 134.94 | -61.47 | 8.72 | 0.19 | 0.709 | 6 |
| StayDur+KDE+Weight+TEMP+Releasetime | 134.95 | -62.47 | 6.7 | 0.244 | 0.675 | 5 |
| KDE+Weight+WDSP+PRCP | 134.96 | -63.48 | 4.69 | 0.321 | 0.658 | 4 |
| ActAvg+KDE+Weight+TEMP+VISIB+Releasetime | 134.97 | -61.49 | 8.68 | 0.192 | 0.704 | 6 |
| StayDur+ActAvg+TEMP+VISIB+PRCP | 134.99 | -62.49 | 6.66 | 0.247 | 0.66 | 5 |
| StayDur+KDE+TEMP+PRCP+Releasetime | 135.05 | -62.53 | 6.6 | 0.252 | 0.679 | 5 |
| StayDur+ActAvg+KDE+Weight+PRCP | 135.06 | -62.53 | 6.59 | 0.253 | 0.664 | 5 |
| StayDur+ActAvg+KDE+Weight+VISIB | 135.07 | -62.53 | 6.58 | 0.253 | 0.66 | 5 |
| StayDur+KDE+Weight+PRCP+Releasetime | 135.11 | -62.55 | 6.54 | 0.257 | 0.675 | 5 |
| ActAvg+Weight+TEMP+VISIB+PRCP | 135.11 | -62.56 | 6.54 | 0.257 | 0.666 | 5 |
| StayDur+ActAvg+KDE+Weight+VISIB+Releasetime | 135.11 | -61.55 | 8.54 | 0.201 | 0.675 | 6 |
| ActAvg+Weight+TEMP+VISIB+PRCP+Releasetime | 135.11 | -61.55 | 8.54 | 0.201 | 0.715 | 6 |
| StayDur+ActAvg+Weight+TEMP+VISIB | 135.12 | -62.56 | 6.53 | 0.258 | 0.664 | 5 |
| StayDur+Weight+TEMP+VISIB+Releasetime | 135.13 | -62.56 | 6.52 | 0.259 | 0.692 | 5 |
| StayDur+TEMP+VISIB+PRCP+Releasetime | 135.13 | -62.57 | 6.52 | 0.259 | 0.703 | 5 |
| StayDur+KDE+Weight+VISIB+Releasetime | 135.14 | -62.57 | 6.51 | 0.26 | 0.672 | 5 |
| StayDur+KDE+Weight+TEMP | 135.17 | -63.58 | 4.48 | 0.345 | 0.642 | 4 |
| StayDur+ActAvg+KDE+VISIB+PRCP | 135.17 | -62.58 | 6.48 | 0.262 | 0.662 | 5 |
| Weight+VISIB | 135.23 | -65.61 | 0.42 | 0.81 | 0.518 | 2 |
| StayDur+ActAvg+Weight+TEMP+PRCP | 135.23 | -62.61 | 6.42 | 0.267 | 0.67 | 5 |
| ActAvg+KDE+Weight+PRCP | 135.25 | -63.62 | 4.41 | 0.354 | 0.653 | 4 |
| StayDur+KDE+VISIB+PRCP+Releasetime | 135.29 | -62.65 | 6.36 | 0.273 | 0.675 | 5 |
| StayDur+KDE+TEMP+VISIB | 135.34 | -63.67 | 4.31 | 0.365 | 0.657 | 4 |
| StayDur+KDE+Weight+PRCP | 135.38 | -63.69 | 4.27 | 0.371 | 0.645 | 4 |
| ActAvg+KDE+VISIB+PRCP | 135.38 | -63.69 | 4.28 | 0.37 | 0.642 | 4 |
| StayDur+ActAvg+Weight+VISIB+PRCP+Releasetime | 135.38 | -61.69 | 8.27 | 0.219 | 0.708 | 6 |
| KDE+VISIB | 135.39 | -65.69 | 0.26 | 0.877 | 0.483 | 2 |
| StayDur+KDE+VISIB+PRCP | 135.4 | -63.7 | 4.25 | 0.373 | 0.647 | 4 |
| KDE+Weight+TEMP+PRCP | 135.4 | -63.7 | 4.25 | 0.373 | 0.623 | 4 |
| KDE+TEMP+VISIB+PRCP | 135.4 | -63.7 | 4.25 | 0.373 | 0.626 | 4 |
| StayDur+KDE+Weight+WDSP+PRCP+Releasetime | 135.41 | -61.71 | 8.24 | 0.221 | 0.685 | 6 |
| StayDur+TEMP+VISIB+PRCP | 135.42 | -63.71 | 4.23 | 0.376 | 0.661 | 4 |
| StayDur+KDE+Weight+VISIB+WDSP+Releasetime | 135.42 | -61.71 | 8.23 | 0.222 | 0.683 | 6 |
| StayDur+Weight+TEMP+PRCP | 135.43 | -63.72 | 4.22 | 0.377 | 0.642 | 4 |
| StayDur+KDE+Weight+TEMP+WDSP+Releasetime | 135.43 | -61.71 | 8.23 | 0.222 | 0.683 | 6 |
| KDE+Weight+TEMP+WDSP | 135.45 | -63.73 | 4.2 | 0.38 | 0.643 | 4 |
| StayDur+Weight+TEMP+PRCP+Releasetime | 135.46 | -62.73 | 6.19 | 0.288 | 0.678 | 5 |
| StayDur+Weight+VISIB+WDSP | 135.48 | -63.74 | 4.17 | 0.384 | 0.682 | 4 |
| StayDur+KDE+Weight+VISIB | 135.49 | -63.75 | 4.16 | 0.385 | 0.653 | 4 |
| StayDur+Weight+TEMP+WDSP | 135.5 | -63.75 | 4.15 | 0.386 | 0.682 | 4 |
| StayDur+KDE+VISIB+WDSP+PRCP+Releasetime | 135.51 | -61.75 | 8.14 | 0.228 | 0.694 | 6 |
| StayDur+KDE+TEMP+WDSP+PRCP+Releasetime | 135.52 | -61.76 | 8.13 | 0.229 | 0.689 | 6 |
| ActAvg+Weight+VISIB+PRCP | 135.53 | -63.77 | 4.12 | 0.39 | 0.658 | 4 |
| StayDur+Weight+VISIB+PRCP+Releasetime | 135.53 | -62.76 | 6.12 | 0.294 | 0.684 | 5 |
| ActAvg+KDE+Weight+VISIB+Releasetime | 135.58 | -62.79 | 6.07 | 0.299 | 0.668 | 5 |
| ActAvg+KDE+Weight+PRCP+Releasetime | 135.6 | -62.8 | 6.06 | 0.301 | 0.666 | 5 |
| StayDur+TEMP+VISIB+WDSP | 135.61 | -63.8 | 4.05 | 0.4 | 0.663 | 4 |
| StayDur+KDE+TEMP+VISIB+WDSP+Releasetime | 135.62 | -61.81 | 8.03 | 0.236 | 0.689 | 6 |
| KDE+Weight+VISIB+WDSP | 135.63 | -63.82 | 4.02 | 0.403 | 0.655 | 4 |
| StayDur+ActAvg+Weight+TEMP+PRCP+Releasetime | 135.63 | -61.82 | 8.02 | 0.237 | 0.687 | 6 |
| StayDur+ActAvg+KDE+Weight+TEMP+VISIB+WDSP+PRCP | 135.64 | -59.82 | 12.01 | 0.151 | 0.726 | 8 |
| KDE+TEMP+VISIB+PRCP+Releasetime | 135.68 | -62.84 | 5.97 | 0.309 | 0.679 | 5 |
| Weight+TEMP+VISIB+PRCP | 135.72 | -63.86 | 3.94 | 0.415 | 0.637 | 4 |
| KDE+TEMP+VISIB+WDSP | 135.73 | -63.87 | 3.92 | 0.417 | 0.628 | 4 |
| StayDur+Weight+TEMP+WDSP+PRCP+Releasetime | 135.73 | -61.87 | 7.92 | 0.244 | 0.703 | 6 |
| StayDur+Weight+VISIB+WDSP+PRCP+Releasetime | 135.73 | -61.87 | 7.92 | 0.244 | 0.705 | 6 |
| Weight+TEMP+VISIB+PRCP+Releasetime | 135.74 | -62.87 | 5.91 | 0.315 | 0.703 | 5 |
| ActAvg+KDE+Weight+TEMP+PRCP+Releasetime | 135.74 | -61.87 | 7.91 | 0.244 | 0.689 | 6 |
| ActAvg+Weight+VISIB+PRCP+Releasetime | 135.75 | -62.88 | 5.9 | 0.316 | 0.674 | 5 |
| StayDur+Weight+TEMP+VISIB+WDSP+Releasetime | 135.76 | -61.88 | 7.89 | 0.246 | 0.701 | 6 |
| StayDur+KDE+VISIB+WDSP+PRCP | 135.77 | -62.89 | 5.88 | 0.318 | 0.683 | 5 |
| ActAvg+KDE+VISIB+PRCP+Releasetime | 135.78 | -62.89 | 5.87 | 0.319 | 0.666 | 5 |
| KDE+Weight+TEMP+VISIB+Releasetime | 135.79 | -62.9 | 5.86 | 0.32 | 0.668 | 5 |
| StayDur+TEMP+VISIB+WDSP+PRCP+Releasetime | 135.79 | -61.9 | 7.86 | 0.249 | 0.705 | 6 |
| KDE+Weight+PRCP | 135.81 | -64.9 | 1.84 | 0.606 | 0.604 | 3 |
| Weight+VISIB+PRCP | 135.82 | -64.91 | 1.83 | 0.609 | 0.629 | 3 |
| StayDur+ActAvg+Weight+VISIB+PRCP | 135.96 | -62.98 | 5.69 | 0.337 | 0.651 | 5 |
| StayDur+Weight+VISIB+PRCP | 135.99 | -63.99 | 3.67 | 0.453 | 0.654 | 4 |
| ActAvg+KDE+Weight+VISIB | 135.99 | -63.99 | 3.67 | 0.453 | 0.632 | 4 |
| KDE+Weight+PRCP+Releasetime | 135.99 | -63.99 | 3.66 | 0.454 | 0.619 | 4 |
| KDE+VISIB+PRCP | 136.01 | -65.01 | 1.64 | 0.651 | 0.611 | 3 |
| Weight+VISIB+PRCP+Releasetime | 136.02 | -64.01 | 3.63 | 0.458 | 0.614 | 4 |
| KDE+Weight+TEMP+WDSP+PRCP+Releasetime | 136.02 | -62.01 | 7.63 | 0.267 | 0.696 | 6 |
| StayDur+KDE+TEMP+WDSP+PRCP | 136.04 | -63.02 | 5.61 | 0.346 | 0.67 | 5 |
| KDE+Weight+TEMP+PRCP+Releasetime | 136.06 | -63.03 | 5.59 | 0.348 | 0.645 | 5 |
| KDE+TEMP+VISIB+WDSP+PRCP+Releasetime | 136.07 | -62.03 | 7.58 | 0.27 | 0.696 | 6 |
| Weight+TEMP+VISIB+WDSP | 136.09 | -64.04 | 3.57 | 0.468 | 0.656 | 4 |
| StayDur+Weight+TEMP+VISIB | 136.1 | -64.05 | 3.55 | 0.47 | 0.644 | 4 |
| StayDur+KDE+Weight+WDSP+PRCP | 136.13 | -63.06 | 5.52 | 0.355 | 0.66 | 5 |
| KDE+Weight+VISIB+WDSP+PRCP+Releasetime | 136.14 | -62.07 | 7.51 | 0.276 | 0.681 | 6 |
| KDE+Weight+VISIB+Releasetime | 136.15 | -64.08 | 3.5 | 0.478 | 0.623 | 4 |
| Weight+TEMP+VISIB+WDSP+PRCP+Releasetime | 136.16 | -62.08 | 7.49 | 0.278 | 0.705 | 6 |
| StayDur+ActAvg+KDE+TEMP+VISIB+PRCP+Releasetime | 136.21 | -61.11 | 9.44 | 0.223 | 0.704 | 7 |
| StayDur+ActAvg+KDE+Weight+TEMP+VISIB+WDSP+PRCP+Releasetime | 136.22 | -59.11 | 13.43 | 0.144 | 0.73 | 9 |
| KDE+TEMP+VISIB+WDSP+PRCP | 136.25 | -63.12 | 5.4 | 0.369 | 0.66 | 5 |
| KDE+Weight+TEMP+VISIB | 136.26 | -64.13 | 3.39 | 0.494 | 0.625 | 4 |
| KDE+VISIB+PRCP+Releasetime | 136.31 | -64.15 | 3.34 | 0.502 | 0.577 | 4 |
| KDE+Weight+TEMP+VISIB+WDSP+Releasetime | 136.31 | -62.15 | 7.34 | 0.29 | 0.691 | 6 |
| KDE+Weight+VISIB+WDSP+PRCP | 136.32 | -63.16 | 5.33 | 0.377 | 0.672 | 5 |
| StayDur+ActAvg+KDE+TEMP+VISIB+PRCP | 136.35 | -62.18 | 7.3 | 0.294 | 0.658 | 6 |
| StayDur+ActAvg+KDE+Weight+TEMP+VISIB | 136.36 | -62.18 | 7.29 | 0.294 | 0.668 | 6 |
| StayDur+KDE+Weight+VISIB+WDSP | 136.37 | -63.19 | 5.28 | 0.383 | 0.696 | 5 |
| KDE+Weight+TEMP+WDSP+PRCP | 136.37 | -63.19 | 5.28 | 0.383 | 0.664 | 5 |
| StayDur+ActAvg+KDE+Weight+TEMP+PRCP | 136.41 | -62.2 | 7.25 | 0.299 | 0.664 | 6 |
| StayDur+Weight+VISIB+WDSP+PRCP | 136.48 | -63.24 | 5.18 | 0.395 | 0.678 | 5 |
| StayDur+TEMP+VISIB+WDSP+PRCP | 136.48 | -63.24 | 5.17 | 0.396 | 0.684 | 5 |
| StayDur+ActAvg+KDE+Weight+TEMP+VISIB+Releasetime | 136.48 | -61.24 | 9.17 | 0.241 | 0.7 | 7 |
| StayDur+KDE+Weight+TEMP+WDSP | 136.55 | -63.28 | 5.1 | 0.404 | 0.677 | 5 |
| StayDur+Weight+TEMP+WDSP+PRCP | 136.61 | -63.3 | 5.05 | 0.41 | 0.678 | 5 |
| StayDur+ActAvg+Weight+TEMP+VISIB+PRCP+Releasetime | 136.63 | -61.31 | 9.03 | 0.251 | 0.717 | 7 |
| StayDur+ActAvg+KDE+Weight+VISIB+PRCP+Releasetime | 136.66 | -61.33 | 8.99 | 0.253 | 0.698 | 7 |
| StayDur+KDE+TEMP+VISIB+WDSP | 136.68 | -63.34 | 4.97 | 0.419 | 0.672 | 5 |
| ActAvg+KDE+Weight+TEMP+VISIB+PRCP | 136.73 | -62.36 | 6.92 | 0.328 | 0.662 | 6 |
| Weight+TEMP+VISIB+WDSP+PRCP | 136.74 | -63.37 | 4.92 | 0.426 | 0.652 | 5 |
| StayDur+ActAvg+KDE+Weight+TEMP+PRCP+Releasetime | 136.75 | -61.38 | 8.9 | 0.26 | 0.689 | 7 |
| StayDur+KDE+Weight+TEMP+PRCP | 136.8 | -63.4 | 4.85 | 0.434 | 0.645 | 5 |
| StayDur+KDE+Weight+TEMP+VISIB+Releasetime | 136.82 | -62.41 | 6.83 | 0.337 | 0.683 | 6 |
| StayDur+KDE+TEMP+VISIB+PRCP | 136.84 | -63.42 | 4.82 | 0.439 | 0.647 | 5 |
| StayDur+KDE+TEMP+VISIB+PRCP+Releasetime | 136.86 | -62.43 | 6.79 | 0.34 | 0.683 | 6 |
| KDE+Weight+VISIB | 136.9 | -65.45 | 0.75 | 0.86 | 0.523 | 3 |
| ActAvg+KDE+Weight+TEMP+VISIB+PRCP+Releasetime | 136.94 | -61.47 | 8.72 | 0.274 | 0.709 | 7 |
| StayDur+KDE+Weight+TEMP+PRCP+Releasetime | 136.95 | -62.47 | 6.7 | 0.349 | 0.674 | 6 |
| StayDur+ActAvg+Weight+TEMP+VISIB+PRCP | 136.98 | -62.49 | 6.67 | 0.352 | 0.672 | 6 |
| StayDur+ActAvg+KDE+Weight+VISIB+PRCP | 137.05 | -62.53 | 6.6 | 0.359 | 0.664 | 6 |
| StayDur+KDE+Weight+VISIB+PRCP+Releasetime | 137.08 | -62.54 | 6.57 | 0.362 | 0.674 | 6 |
| StayDur+Weight+TEMP+VISIB+PRCP+Releasetime | 137.12 | -62.56 | 6.53 | 0.367 | 0.699 | 6 |
| StayDur+KDE+Weight+TEMP+VISIB | 137.17 | -63.58 | 4.48 | 0.482 | 0.642 | 5 |
| ActAvg+KDE+Weight+VISIB+PRCP | 137.21 | -63.6 | 4.45 | 0.487 | 0.658 | 5 |
| StayDur+KDE+Weight+VISIB+PRCP | 137.23 | -63.62 | 4.42 | 0.491 | 0.653 | 5 |
| StayDur+KDE+Weight+TEMP+VISIB+WDSP+Releasetime | 137.38 | -61.69 | 8.27 | 0.309 | 0.689 | 7 |
| StayDur+KDE+Weight+VISIB+WDSP+PRCP+Releasetime | 137.38 | -61.69 | 8.27 | 0.309 | 0.687 | 7 |
| KDE+Weight+TEMP+VISIB+PRCP | 137.39 | -63.69 | 4.26 | 0.512 | 0.623 | 5 |
| KDE+Weight+TEMP+VISIB+WDSP | 137.4 | -63.7 | 4.25 | 0.514 | 0.638 | 5 |
| StayDur+KDE+Weight+TEMP+WDSP+PRCP+Releasetime | 137.41 | -61.71 | 8.24 | 0.312 | 0.683 | 7 |
| StayDur+Weight+TEMP+VISIB+PRCP | 137.42 | -63.71 | 4.23 | 0.517 | 0.654 | 5 |
| StayDur+Weight+TEMP+VISIB+WDSP | 137.48 | -63.74 | 4.17 | 0.525 | 0.682 | 5 |
| StayDur+KDE+TEMP+VISIB+WDSP+PRCP+Releasetime | 137.51 | -61.75 | 8.14 | 0.32 | 0.694 | 7 |
| ActAvg+KDE+Weight+VISIB+PRCP+Releasetime | 137.56 | -62.78 | 6.09 | 0.413 | 0.67 | 6 |
| KDE+Weight+VISIB+PRCP | 137.63 | -64.82 | 2.02 | 0.733 | 0.623 | 4 |
| StayDur+KDE+Weight+VISIB+WDSP+PRCP | 137.67 | -62.83 | 5.98 | 0.425 | 0.685 | 6 |
| KDE+Weight+TEMP+VISIB+PRCP+Releasetime | 137.68 | -62.84 | 5.97 | 0.426 | 0.677 | 6 |
| StayDur+Weight+TEMP+VISIB+WDSP+PRCP+Releasetime | 137.73 | -61.86 | 7.92 | 0.339 | 0.703 | 7 |
| StayDur+KDE+TEMP+VISIB+WDSP+PRCP | 137.77 | -62.88 | 5.88 | 0.436 | 0.679 | 6 |
| KDE+Weight+VISIB+PRCP+Releasetime | 137.99 | -63.99 | 3.66 | 0.599 | 0.619 | 5 |
| StayDur+KDE+Weight+TEMP+WDSP+PRCP | 138 | -63 | 5.65 | 0.464 | 0.666 | 6 |
| KDE+Weight+TEMP+VISIB+WDSP+PRCP+Releasetime | 138.02 | -62.01 | 7.63 | 0.366 | 0.694 | 7 |
| StayDur+ActAvg+KDE+Weight+TEMP+VISIB+PRCP+Releasetime | 138.15 | -61.07 | 9.5 | 0.302 | 0.709 | 8 |
| KDE+Weight+TEMP+VISIB+WDSP+PRCP | 138.18 | -63.09 | 5.48 | 0.484 | 0.666 | 6 |
| StayDur+ActAvg+KDE+Weight+TEMP+VISIB+PRCP | 138.33 | -62.17 | 7.32 | 0.396 | 0.657 | 7 |
| StayDur+KDE+Weight+TEMP+VISIB+WDSP | 138.37 | -63.18 | 5.29 | 0.508 | 0.696 | 6 |
| StayDur+Weight+TEMP+VISIB+WDSP+PRCP | 138.47 | -63.24 | 5.18 | 0.521 | 0.678 | 6 |
| StayDur+KDE+Weight+TEMP+VISIB+PRCP | 138.79 | -63.4 | 4.86 | 0.562 | 0.642 | 6 |
| StayDur+KDE+Weight+TEMP+VISIB+PRCP+Releasetime | 138.81 | -62.4 | 6.84 | 0.445 | 0.685 | 7 |
| StayDur+KDE+Weight+TEMP+VISIB+WDSP+PRCP+Releasetime | 139.35 | -61.68 | 8.3 | 0.405 | 0.692 | 8 |
| StayDur+KDE+Weight+TEMP+VISIB+WDSP+PRCP | 139.67 | -62.83 | 5.98 | 0.542 | 0.685 | 7 |
